# Eurocan plus report: feasibility study for coordination of national cancer research activities

**DOI:** 10.3332/ecancer.2011.84

**Published:** 2008-05-20

## Abstract

The EUROCAN+PLUS Project, called for by the European Parliament, was launched in October 2005 as a feasibility study for coordination of national cancer research activities in Europe. Over the course of the next two years, the Project process organized over 60 large meetings and countless smaller meetings that gathered in total over a thousand people, the largest Europe–wide consultation ever conducted in the field of cancer research.

Despite a strong tradition in biomedical science in Europe, fragmentation and lack of sustainability remain formidable challenges for implementing innovative cancer research and cancer care improvement. There is an enormous duplication of research effort in the Member States, which wastes time, wastes money and severely limits the total intellectual concentration on the wide cancer problem. There is a striking lack of communication between some of the biggest actors on the European scene, and there are palpable tensions between funders and those researchers seeking funds.

It is essential to include the patients’ voice in the establishment of priority areas in cancer research at the present time. The necessity to have dialogue between funders and scientists to establish the best mechanisms to meet the needs of the entire community is evident. A top priority should be the development of translational research (in its widest form), leading to the development of effective and innovative cancer treatments and preventive strategies. Translational research ranges from bench–to–bedside innovative cancer therapies and extends to include bringing about changes in population behaviours when a risk factor is established.

The EUROCAN+PLUS Project recommends the creation of a small, permanent and independent European Cancer Initiative (ECI). This should be a model structure and was widely supported at both General Assemblies of the project. The ECI should assume responsibility for stimulating innovative cancer research and facilitating processes, becoming the common voice of the cancer research community and serving as an interface between the cancer research community and European citizens, patients’ organizations, European institutions, Member States, industry and small and medium enterprises (SMEs), putting into practice solutions aimed at alleviating barriers to collaboration and coordination of cancer research activities in the European Union, and dealing with legal and regulatory issues. The development of an effective ECI will require time, but this entity should be established immediately. As an initial step, coordination efforts should be directed towards the creation of a platform on translational research that could encompass (1) coordination between basic, clinical and epidemiological research; (2) formal agreements of co–operation between comprehensive cancer centres and basic research laboratories throughout Europe and (3) networking between funding bodies at the European level.

The European Parliament and its instruments have had a major influence in cancer control in Europe, notably in tobacco control and in the implementation of effective population–based screening. To make further progress there is a need for novelty and innovation in cancer research and prevention in Europe, and having a platform such as the ECI, where those involved in all aspects of cancer research can meet, discuss and interact, is a decisive development for Europe.

## Executive Summary

Cancer is one of the biggest public health crises facing Europe in the 21st century—one for which Europe is currently not prepared nor preparing itself. Cancer is a major cause of death in Europe with two million casualties and three million new cases diagnosed annually, and the situation is set to worsen as the population ages.

These facts led the European Parliament, through the Research Directorate-General of the European Commission, to call for initiatives for better coordination of cancer research efforts in the European Union. The EUROCAN+PLUS Project was launched in October 2005 as a feasibility study for coordination of national cancer research activities. Over the course of the next two years, the Project process organized over 60 large meetings and countless smaller meetings that gathered in total over a thousand people. In this respect, the Project became the largest Europe-wide consultation ever conducted in the field of cancer research, implicating researchers, cancer centres and hospitals, administrators, healthcare professionals, funding agencies, industry, patients’ organizations and patients.

The Project first identified barriers impeding research and collaboration in research in Europe. Despite a strong tradition in biomedical science in Europe, fragmentation and lack of sustainability remain the formidable challenges for implementing innovative cancer research and cancer care improvement. There is an enormous duplication of research effort in the Member States, which wastes time, wastes money and severely limits the total intellectual concentration on the wide cancer problem. There is a striking lack of communication between some of the biggest actors on the European scene, and there are palpable tensions between funders and those researchers seeking funds.

In addition, there is a shortage of leadership, a multiplicity of institutions each focusing on its own agenda, sub–optimal contact with industry, inadequate training, non–existent career paths, low personnel mobility in research especially among clinicians and inefficient funding—all conspiring against efficient collaboration in cancer care and research. European cancer research today does not have a functional translational research continuum, that is the process that exploits biomedical research innovations and converts them into prevention methods, diagnostic tools and therapies. Moreover, epidemiological research is not integrated with other types of cancer research, and the implementation of the European Directives on Clinical Trials 1 and on Personal Data Protection 2 has further slowed the innovation process in Europe. Furthermore, large inequalities in health and research exist between the EU–15 and the New Member States.

The picture is not entirely bleak, however, as the European cancer research scene presents several strengths, such as excellent basic research and clinical research and innovative etiological research that should be better exploited.

When considering recommendations, several priority dimensions had to be retained. It is essential that proposals include actions and recommendations that can benefit all Member States of the European Union and not just States with the elite centres. It is also essential to have a broader patient orientation to help provide the knowledge to establish cancer control possibilities when we exhaust what can be achieved by the implementation of current knowledge. It is vital that the actions proposed can contribute to the Lisbon Strategy to make Europe more innovative and competitive in (cancer) research.

The Project participants identified six areas for which consensus solutions should be implemented in order to obtain better coordination of cancer research activities. The required solutions are as follows.

1. The proactive management of innovation, detection, facilitation of collaborations and maintenance of healthy competition within the European cancer research community.
2. The establishment of an exchange portal of information for health professionals, patients and policy makers.
3. The provision of guidance for translational and clinical research including the establishment of a translational research platform involving comprehensive cancer centres and cancer research centres.
4. The coordination of calls and financial management of cancer research projects.
5. The construction of a ‘one–stop shop’ as a contact interface between the industry, small and medium enterprises, scientists and other stakeholders.
6. The support of greater involvement of healthcare professionals in translational research and multidisciplinary training.

In the course of the EUROCAN+PLUS consultative process, several key collaborative projects emerged between the various groups and institutes engaged in the consultation. There was a collaboration network established with Europe’s leading Comprehensive Cancer Centres; funding was awarded for a closer collaboration of Owners of Cancer Registries in Europe (EUROCOURSE); there was funding received from FP7 for an extensive network of leading Biological Resource Centres in Europe (BBMRI); a Working Group identified the special needs of Central, Eastern and South–eastern Europe and proposed a remedy (‘Warsaw Declaration’), and the concept of developing a one–stop shop for dealing with academia and industry including the Innovative Medicines Initiative (IMI) was discussed in detail.

Several other dimensions currently lacking were identified. There is an absolute necessity to include the patients’ voice in the establishment of priority areas in cancer research at the present time. It was a salutary lesson when it was recognized that all that is known about the quality of life of the cancer patient comes from the experience of a tiny proportion of cancer patients included in a few clinical trials. The necessity to have dialogue between funders and scientists to establish the best mechanisms to meet the needs of the entire community was evident. A top priority should be the development of translational research (in its widest form) and the development of effective and innovative cancer treatments and preventative strategies in the European Union. Translational research ranges from bench-to-bedside innovative cancer therapies and extends to include bringing about changes in population behaviours when a risk factor is established.

Having taken note of the barriers and the solutions and having examined relevant examples of existing European organizations in the field, it was agreed during the General Assembly of 19 November 2007 that the EUROCAN+PLUS Project had to recommend the creation of a small, permanent and neutral ECI. This should be a model structure and was widely supported at both General Assemblies of the project. The proposal is based on the successful model of the European Molecular Biology Organisation (EMBO), and its principal aims include providing a forum where researchers from all backgrounds and from all countries can meet with members of other specialities including patients, nurses, clinicians, funders and scientific administrators to develop priority programmes to make Europe more competitive in research and more focused on the cancer patient.

The ECI should assume responsibility for:

1. stimulating innovative cancer research and facilitating processes;
2. becoming the common voice of the cancer research community and serving as an interface between the cancer research community and European citizens, patients’ and organizations;
3. European institutions, Member States, industry and small and medium enterprises;
4. putting into practice the aforementioned solutions aimed at alleviating barriers and coordinating cancer research activities in the EU;
5. dealing with legal and regulatory issues.

Solutions implemented through the ECI will lead to better coordination and collaboration throughout Europe, more efficient use of resources, an increase in Europe’s attractiveness to the biomedical industry and better quality of cancer research and education of health professionals.

The Project considered that European legal instruments currently available were inadequate for addressing many aspects of the barriers identified and for the implementation of effective, lasting solutions. Therefore, the legal environment that could shelter an idea like the ECI remains to be defined but should be done so as a priority. In this context, the initiative of the European Commission for a new legal entity for research infrastructure might be a step in this direction. The development of an effective ECI will require time, but this should be established immediately. As an initial step, coordination efforts should be directed towards the creation of a platform on translational research that could encompass: (1) coordination between basic, clinical and epidemiological research; (2) formal agreements of co-operation between comprehensive cancer centres and basic research laboratories throughout Europe; (3) networking between funding bodies at the European level. Another topic deserving immediate attention is the creation of a European database on cancer research projects and cancer research facilities.

Despite enormous progress in cancer control in Europe during the past two decades, there was an increase of 300,000 in the number of new cases of cancer diagnosed between 2004 and 2006. The European Parliament and its instruments have had a major influence in cancer control, notably in tobacco control and in the implementation of effective population–based screening. To make further progress there is a need for novelty and innovation in cancer research and prevention in Europe, and having a platform such as the ECI, where those involved in all aspects of cancer research can meet, discuss and interact, is a decisive development for Europe.

## Contents

1. Rationale for the Eurocan+Plus Project
2. Objective of the Eurocan+Plus Project
3. The Eurocan+Plus process
4. Contractual assignments and directions taken
5. Advantages of biomedical research in Europe
6. Recognized barriers impeding care and research in Europe
  6.1. Lack of a global vision and leadership in the cancer research community
  6.2. Lack of a translational research process on the European level
  6.3. Lack of integration of epidemiological research with other types of research
  6.4. Legal and administrative barriers to translational research and to epidemiological research and surveillance
  6.5. Lack of functional contact with industry
  6.6. Lack of functional information flow
  6.7. Lack of mobility in research
  6.8. Lack of efficient funding
  6.9. Lack of efficient networking
7. Six agreed–upon solutions to surmount barriers to coordination
  7.1. Proactive management of innovation and facilitation of collaborations while maintaining an atmosphere of healthy competition within the European cancer community
  7.2. Establishment of an information exchange portal (brokerage of knowledge) for health professionals, patients and policy makers
  7.3. The provision of guidance for translational and clinical research including the establishment of a translational research platform involving comprehensive cancer centres and cancer research centres
  7.4. Coordination of calls and financial management for cancer research projects
  7.5. The construction of a ‘one–stop shop’ as a contact interface for industry, small and medium enterprises, scientists and other stakeholders
  7.6. Support for greater involvement of health professionals in translational research and multidisciplinary training
8. Recommendations of the Eurocan+Plus Project
9. Proposal for an European Cancer Initiative (ECI)
  9.1. How the ECI would work
  9.2. ECI Functioning
  9.3. ECI Organisation
10. Short–term plans
11. Examples of European organizations active in cancer research
  11.1. TuBaFrost
  11.2. National Cancer Research Institute (NCRI)
  11.3. European Molecular Biology Organization (EMBO)
  11.4. European Strategy Forum on Research Infrastructures (ESFRI)
  11.5. Innovative Medicines Initiative (IMI)
  11.6. Coordination of Cancer Clinical Practice Guidelines in Europe (CoCanCPG)
  11.7. Other examples of European co–operation

## Foreword

The process that resulted in the submission and funding of the Eurocan+Plus Project (hereafter termed ‘the Project’) by the 6th Framework Programme was initiated by the European Parliament.

This report summarizes the key findings and conclusions of the Eurocan+Plus Project, which ran between October 2005 and December 2007, and outlines proposals for action in the short and the longer term.

Participants in the Project represented themselves and not the institution where they work. The Project was in no way a formal collaboration between any governmental body, funding agency, research or medical institution of any of the 27 EU Member States. In this respect, proposals in this Summary Report and in other deliverables in no way represent a formal commitment of any governmental or non–governmental institution for any idea proposed by the Project. For more information on the topics developed in this report, interested readers are invited to consult the reports issued by the different Work Packages. These reports can be consulted on the Project website (www.eurocanplus.eu) or can be obtained from the Project Coordination at eurocan@iarc.fr.

Work Package leaders and specific sub–group leaders

WP1: Project coordination: Peter Boyle, International Agency for Research on Cancer, Lyon
WP2: Coordination of Part I (including WP2 to WP7): Thomas Tursz, Institut Gustave Roussy, Paris
WP3: Analysis of barriers to collaboration: Thomas Tursz
  Specific themes investigated by WP3 sub–groups:
  1. Clinical cancer research: Thomas Tursz
  2. Basic cancer research: Julio E. celis, Danish Cancer Society, Copenhagen
  3. Translational cancer research: David Kerr, The Chancellor, Masters and Scholars of the University of Oxford, Oxford
  4. Cancer prevention research: Jose M. Martin–Moreno, Universidad de Valencia Facultad de Medicina y Odontologia, Valencia
  5. Cancer aetiology research: Dimitrios Trichopoulos, National and Kapodistrian University of Athens, Athens
  6. Research in specific populations: Herbert Michael Pinedo and Winald Gerritsen, VUmc Cancer Center, Amsterdam
  7. Survival potential: Harry Bartelink, The Netherlands Cancer Institute, Amsterdam
  8. New approaches to biotherapies: Alexander M. M. Eggermont, Erasmus University Medical Center, Rotterdam
  9. Migrant populations: Hans Henrik Storm, Danish Cancer Society, Copenhagen
WP4: Requirements in research on quality control to increase survival: Diana Dunstan, Medical Research Council, London
WP5: Evaluation and integration of national cancer plans: John M. Fitzpatrick, University College Dublin, Dublin, and Renée Otter, Comprehensive Cancer Center Groeningen, Groeningen
WP6: Regulatory and ethical guidelines and critical issues for coordination of clinical trials of therapy and immune prevention of cancer: Filippo Belardelli, Istituto Superiore di Sanita, Rome, and Dominique de Valeriola, Jules Bordet Institute, Brussels
WP7: Guidelines for translational research, certifying laboratories: Julio E. Celis, Danish Cancer Society, Copenhagen
WP8: Coordination of Part II, Management and partner recruitment (including WP8 to WP11): Peter Lange, Bundesministerium fuer Bildung und Forschung, Berlin
WP9: Funding of cancer research in the 25 EU Member States and in countries with an R&D affiliation with the EU: Jan–Wilhelm Hartgerink, Ministerie van Volksgezondheid, Welzjin en Sport, Den Haag, and Edvard BEEM, Netherlands Organization for Health Research and Development (ZonMW), Amsterdam
WP10: Governance, legal and financial structure of an European economic interest group: Ulrik Ringborg, Karolinska Institutet, Stockholm
WP11: Developing public–private partnership and collaboration with industry: David Kerr, The Chancellor, Masters and Scholars of the University of Oxford, Oxford

Cross cutting issues (attached to WP1): mobility of researchers in Europe: Markus Pasterk and Hemma Bauer, Ministry of Education, Science and Culture, Vienna; cancer research in the new Member States: Witold ZATONSKI, Maria Sklodowska–Curie Memorial Cancer Center and Institute of Oncology, Warsaw.

Project coordination team (attached to WP1): Philippe Autier, IARC, Lyon; Asiedua Asante, IARC, Lyon; Jean–Pierre Boissel, Claude Bernard University, Lyon; Jean–François Démonet, INSERM, Lyon; Caroline Granger, IARC, Jan Alvar Lindencrona (attached to WP10), Karolinska Institutet, Stockholm; Gordon McVie, European Cancer Institute, Milan. This summary report was drafted by Philippe Autier and Jan–Alvar Lindencrona.

The complete list of participants is available at www.eurocanplus.eu

## 1. Rationale for the Eurocan+Plus Project

Science and technology are playing a steadily growing role in the wealth of nations and in the alleviation of suffering from disease. Cancer is a particularly unique challenge, as it represents a heterogeneous group of diseases comprising about 200 different types of malignant conditions that can affect all organs. Different cancers have different risk factors, different biological and clinical courses and require different treatments.

Cancer has a dominant position in causes of death in Europe, as it is responsible for 25% of all deaths and is the biggest killer of people aged 45–64 [2].

The cancer burden is set to increase as the European population is aging. In 2006, 2.2 million people in the EU–27 were diagnosed with a cancer, and in 2020, just by virtue of aging, three million will be diagnosed with a cancer [3]. In 2008, differences in access to screening and to modern treatments still exist between the EU–15 and the new Member States (the EU–12), although improvements have been seen in some Member States over the past decade.

Member States generally have their own research policies and structures, quite often of a high standard, but on an European level there is fragmentation and inefficient use of resources. To offset this perilous situation, in March 2000, the EU Heads of States and Governments agreed to make the EU ‘the most competitive and dynamic knowledge–driven economy by 2010’, by creating the European Research Area (ERA). This agreement and the objective of devoting 3% of gross domestic product to research were called the Lisbon Agenda. Although some progress was made towards innovating Europe’s research scene, there is growing concern that the reform process in science and technology is not going fast enough, and that the ambitious targets will not be reached.

Until the beginning of the 1990s, Europe had a strong position in biomedical research and most pharmaceutical companies maintained large research facilities in Europe. It was estimated that in the 1980s, around two–thirds of pharmaceutical research was done in Europe. This success in an investigative context was echoed by the far–reaching Europe Against Cancer Programme that was discontinued in the 1990s. Many new drugs were tested through phase I, II and III trials [4] in European settings. During the 1990s, biomedical companies initiated large transfers of resources and competencies out of Europe to North America and Asia, which has threatened the competitive position of Europe in biomedical research. As a consequence, development of new treatments and of new detection or prevention methods depend more and more on research performed outside Europe.

The reasons why many research activities are transferred outside Europe are well known [5]. Europe plays a leading world role in terms of scientific excellence but largely fails to convert science–based findings and inventions into innovative products and services capable of boosting competitiveness. For many years, Europe has been unable to retain many of its most talented scientists and is unable to provide a scientific environment capable of attracting top young scientists trained elsewhere. Since 2000, the brain drain traditionally exerted by the USA has been aggravated by Asian countries that have started attracting brilliant and experienced European researchers.

Public and patient’s expectations from public health and medical research are huge. An Eurobarometer survey in 2007 showed that the majority of Europeans are interested in medical and health research (71%), as well as in science and technology (60%) and 60% are mostly interested in new discoveries in medicine [6]. This means it is time for Europe to decide how to stimulate and maintain creativity and innovation in cancer research in today’s globally competitive environment and on retaining and attracting promising scientists.

The Framework Programmes (FPs) of the European Commission (specially the 6th FP) were expected to support the networking of cancer research efforts in the EU. It appeared however, that the cancer world had genuine characteristics that made such networking difficult, for instance the complexity of cancer and of cancer research, and the numerous institutions and groups active in the cancer field. Therefore, it was felt that cancer deserved a specific approach. In this respect, the Eurocan+Plus Project (hereafter referred to as ‘the Project’) can be viewed as an invitation to the European cancer community to propose solutions of its own, influencing its own destiny and improving cancer research in Europe, all ultimately for the benefit of cancer patients.

## 2. Objective of the Eurocan+Plus Project

The principal objective of the Project was the definition of the best ways to improve cancer research in Europe through better coordination of stakeholders.

Better coordination is deemed to have positive effects on:

- efficient use of resources;
- quality of cancer research;
- quality of care;
- attractiveness of Europe for biomedical industry;
- education of health professionals as well as of patients.

## 3. The Eurocan+Plus process

The Project started in October 2005 and ended in December 2007. In two years, the Project process organized over 60 large meetings and countless smaller meetings, gathering in total over a thousand people. A key achievement of the Project was to enable discussions, confrontation of views and circulation of working documents between the multiple stakeholders in cancer research, many of whom had never had such an opportunity before the Project. As a result, most participants made significant efforts to overcome their own views and agendas so as to build a common vision for the European cancer research community. The Project was structured around 11 work packages (WP) that covered most scientific, human, financial and legal areas relevant to cancer research in Europe. The Project has identified and described barriers to collaboration in all domains of cancer research in Europe. This was the specific task of several work packages included in Part I (WPs 2 to 7).

Part II (WPs 8 to 11) of the project has evaluated and identified solutions related to functional aspects of the cancer research process which should be co–ordinated at the European level for improving performance and efficiency in the EU, split into six serviceable areas. The first General Assembly of December 2006 issued a number of practical proposals for tackling barriers in cancer research, which were worked on during the second year of the Project. Then the Project team examined the best structure to ensure the sustainable implementation of these solutions in the long term. The search for the most adequate coordination modalities took into consideration already—or nearly—existing coordinating structures, infrastructures and initiatives in the biomedical field in Europe that are generally viewed as being relevant and useful (see Chapter 11).

All work performed, including meetings organized by the project, has been carried out with the following caveats.

- Be inclusive and not exclusive, while ensuring that all decisions were reached with the aim of fulfilling the Project’s objectives.
- Include all stakeholders concerned with cancer research in Europe:
  – Clinical health professionals;
  – Researchers in basic science;
  – Scientists, health professionals and civil servants involved in prevention, screening, public health and epidemiology;
  – Representatives of governmental and non–governmental funding agencies;
  – Representatives of the biomedical industry;
  – Patients’ organizations;
  – Representatives of the European Commission;
  – Members of the European Parliament;
  – Representatives of Governments of Member States.
- Complement and integrate in its process already existing coordinated activities in the EU, for example:
  – The European Association for Cancer Research (EACR).
  – The European Cancer Organisation (ECCO).
  – The European Life Sciences Forum (ELSF).
  – The European Molecular Biology Organisation (EMBO).
  – The European Organisation for Research and Treatment of Cancer (EORTC).
  – The Federation of European Biochemical Societies (FEBS).
  – The Initiative for Science in Europe (ISE).
  – The Organisation of European Cancer Institutes (OECI).
  – The Patients’ organizations (e.g. the European Cancer Patient Coalition (ECPC), Europa Donna, Europa Uomo).
- Complement other EU projects and policies on Science and Technology, for example:
  – The European Strategy Forum on Research Infrastructures (ESFRI).
  – The Innovative Medicines Initiative (IMI).
  – The project for Coordination of Cancer Clinical Practice Guidelines in Europe (CoCanCPG).
  – The European Research Council (ERC).
- Complement other ‘diagnosis reports’ on Science and Technology in the EU (e.g. the Green Paper and its annexes) [7]. In this respect, a number of issues addressed hereafter are relevant to other segments of biomedical research in Europe.

## 4. Contractual assignments and directions taken

The Annex I entitled ‘Description of the Work’ of the contract No. LSSC–CT–2005–015197 stipulated six objectives for the proposed Specific Support Action. Objective 1 was ‘to identify the fields, topics and research subjects where the lack of coordination of national activities is particularly detrimental for the progress of knowledge and quality of care’ [8]. Extensive consultations and discussions that took place during the Project brought a radical change in perspective to this first objective.

The Project found that the problem of fragmentation of cancer research that is mainly due to the coexistence of a multiplicity of institutions and players prevails in most European countries, and thus the lack of coordination of cancer research has an essentially European dimension. Thus issues addressed by the Project are not especially linked to ‘lack of coordination of national activities’, but rather to lack of coordination at the European level. As a matter of fact, most European countries cannot accrue the scientific critical mass (see further for a definition) needed for world–class research in all domains. For Project participants, the detrimental effects of lack of coordination are chiefly related to European rather than to national issues. This opinion largely agrees with the Eurobarometer survey done in 2005 showing that 88% of Europeans believe that researchers in different European countries should co–operate more with each other, and 83% consider that there should be more coordination of research between the Member States of the European Union [9].

About the ‘fields, topics and research subjects for which lack of coordination would be particularly detrimental’, the Project will issue proposals of priority research topics for which better coordination is warranted through (for instance) partnerships between institutions involved in basic, clinical, epidemiological and translational research [10]. Multiple discussions between a large variety of scientists in basic, clinical and public health addressed this question, and all concluded that true innovation and genuine breakthrough is not the consequence of centrally planed research activities focused on particular areas, but rather the result of encouraging creativity and (risky) innovations and fostering connections between the various research areas. Significant discovery may come from any research area and a discovery in one area may have considerable influence in a remote area. Hence, the Project found that the problem is not really the lack of coordination of research activities in specific areas, but that all is about encouraging innovation (including ‘blue sky’ research) and building bridges between very diverse research domains, without *a priori* knowledge about where significant discoveries will arise.

## 5. Advantages of biomedical research in Europe

The Eurocan+Plus Project revealed that the picture of biomedical research in Europe is not so bleak, and that compared to the USA and Asia, the European scene still has a number of positive qualities, among which are:

- While phase III trials seem likely to migrate to Asia (mainly because of cost and future market considerations), Europe remains excellent for organizing phase I and II clinical trials; this should remain a niche for most large cancer centres.
- European cancer centres have interesting biobanking resources still unmatched by their American or Asian counterparts.
- About 5% of all cancer patients in Europe are included in clinical trials: this may appear to be a low figure, but it is better than in the USA, where clinical research institutions compete with private oncology centres for patients.
- The scientific output for each Euro invested in cancer research is larger in Europe than in the USA.
- Most European countries are now equipped with cancer registries, which they should exploit for evaluating new treatments and translational research with long-term follow-up. As an example on ways to strengthen European scientific assets, an ERANET project called EUROCOURSE has now been selected by the 7th Framework Programme for improving the use of European Cancer Registries for cancer research.
- Networking initiatives in the domain of leukaemia have existed for over 30 years. Currently, more than 1000 physicians and researchers cooperate in 141 institutions in 26 countries caring for over 10,000 leukaemia patients Europe-wide (www.leukemianet.org) [11]. In 2002, European leukaemia researchers in close co-operation with interdisciplinary research partners and industry embarked on an Europe-wide network, the European Leukemia Net (ELN), which includes all major European leukaemia study groups and their interdisciplinary partner groups. Other networking initiatives have also been successful in the cancer domain, for instance in molecular imaging (www.emilnet.org) or in the genetics of melanoma (www.genomal.org).
- Successful coordination of research activities and of other innovative actions already exists in several domains, for instance:
  – The coordination of funds from charities by the National Cancer Research Initiative (NCRI) in the United Kingdom represents a model of efficient funding for cancer research activities.
  – The creation of a network of cancer institutes in Italy under the auspices of the Istituto Superiore di Sanita (ISS), the major governmental funding agency for health research in Italy, is fruitful.
  – In several countries, individual cancer institutions have acquired world–class clinical research components, for example in the United Kingdom, the Netherlands and Sweden; co–operation on occasional projects has worked well.
  – Several countries have organized successful special training programmes and career curricula for health professionals more involved in applied research activities (e.g. the MD/PhD programmes in the Netherlands, the United Kingdom and Sweden).

## 6. Recognized barriers impeding care and research in Europe

The barriers outlined in this chapter reflect the consensus view of Project participants.

### 6.1. Lack of a global vision and leadership in the cancer research community

Many attempts have been made to coordinate research and researchers in Europe. Most of them have been either local or oriented around a specific discipline or disease. Fragmentation and duplication is a problem common to many research activities in Europe, contributing to wasting human and financial resources, decreasing the possibility of gathering the critical scientific mass (see definition later) necessary to perform sustainable world–class research and increasing the duration of the discovery process [12]. Reviews of the Framework Programmes have noticed that these programmes were not able to combat fragmentation [13]. People who work in cancer research represent a heterogeneous group, each with his or her own vision, agenda and overlapping memberships. If cancer research is complex, the cancer community in Europe is complex too, so much so that external observers have considerable difficulties in appraising issues at stake in cancer research. This situation also prevails at the country level.

This lack of a single voice is a barrier to collaboration. The lack of a common voice for the European cancer research community is detrimental for fruitful interactions with policy makers, administrations, industry, health professional organizations and patients’ organizations. Industry, policy makers and institutions are growing weary of not having a permanent discussion partner representing the cancer research community as a whole. The Commission has launched two agencies, the European Food Safety Agency (EFSA) for food safety and the European Centre for Disease Control (ECDC) for epidemic intelligence, which has led to a revival of these fields, but there is no such single European agency for cancer. Instead we have a myriad of individuals, institutions, networks and small groups, each with their own agenda and multiple memberships. Although most are admirable in trying to focus on their specific view of cancer research, there is no global vision.

Lack of such a vision and the lack of a single voice make it hard for the cancer research community to swiftly answer emerging needs and invest promptly in novel research ideas, not to mention correcting the misinformation that plagues the media.

Yet, if we are to have an efficient plan for cancer research in Europe, we must have an overall vision and a common body that can speak for all stakeholders and take into account all of their views. In contrast, the US National Cancer Institute has led an extramural programme since the National Cancer Act of 1971, which has given not only stability, but leadership, coordination and innovation to the country as well. Also, the US–NCI did not strangle the voice of other prominent US organizations (e.g. AACR, ASCO, ACS) but rather served as a focal point for them. Only a handful of member states have developed National Cancer Plans. These are uneven with regards to content and implementation. For instance, only two countries (France and the United Kingdom) have evaluated their cancer plans, which mean that we have no way of knowing if the cancer plans in Europe are effective or not. A general European framework of criteria for cancer plans would be a valuable addition to existing plans and would serve as a guideline for countries wishing to establish one.

### 6.2. Lack of a translational research process on the European level

#### 6.2.1. Defining translational research

Translational research is the process by which a concept or discovery (e.g. new molecule, new process, new imaging technology) from basic science is transformed into a product that can be used in the clinic [14]. This is quite different from the drug–discovery method which starts by screening and testing large numbers of molecules, from which potential new therapies are selected for so–called Phase I clinical trials (assessment of dose and of toxicity in humans) [15].

#### 6.2.2. Integrated translational research

Integrated translational research can be defined as a hard–wired process that leads basic, clinical and epidemiological researchers to work together to solve research problems. Unfortunately, such a process is still rare in the cancer domain in Europe despite a consensus that there is a need to capitalise on the potential of basic research discoveries in clinical usage. The Project has identified many examples of world–class basic research centres in Europe that experience considerable difficulty accessing and collaborating with hospitals.

The main reasons for this lack of integration are:

1. Most European academic institutions seldom gather the necessary intellectual resources, technical platform, access to patients and coordination capacity necessary for achieving the scientific critical mass needed to perform extensive, long–term research on new technologies or treatment modalities from basic research to delivery to patients [16].
2. Basic science is funded by governments and charities while clinical trials are most often paid for by industry. Translational research receives little attention from funders. In many countries, the costs of translational research efforts are in fact covered by healthcare reimbursements from social security agencies or insurance. There are some exceptions, however, for instance, the United Kingdom, Sweden and the Netherlands have developed efficient structures for early testing of new anti–cancer agents, and their experiences may serve as examples for the rest of Europe. To fill in this gap, the 7th Framework Programme of the EU has among its objectives for the health domain for the period 2007–2013 the support of translational research projects. It remains to be seen whether this support will translate into effective world–class projects in the cancer field.
3. The current career system in most academic institutions/hospitals does not favour translational research projects, which are expensive, often take years to conduct and will not result in articles published in high–impact–factor scientific journals (journals dedicated to basic science have higher impact factors). This means translational research is not attractive to basic researchers, who feel that it will not greatly advance their careers, and it is difficult to recruit clinicians who are trained in translational research since the career systems in most hospitals do not favour involvement in early proof–of–concept clinical trials.
4. Many healthcare institutions are struggling for money, and patient care takes the lion’s share of the available human resources, leaving little time or space to perform translational research in cancer wards and clinics.
5. Efficient translational research requires excellence in basic and clinical research as well as the possibility and the ability to perform long–term patient follow–up (e.g. though cancer registries). Regrettably, there is a serious shortage of clinicians involved in research programmes, and university hospitals have difficulty recruiting and retaining top clinical scientists. The gap in finding clinicians skilled in research and able to devote time to translational projects is true in all fields of cancer research, but especially troublesome in certain disciplines like radiology and pathology, in particular molecular pathology. Europe already faces a severe recruiting problem that will worsen in the next decade as academics in these areas retire.
6. Marketing departments of biomedical industries heavily fund clinical studies at the national level to promote their products and not for real scientific objectives or phase I, II and III trials. This way of funding diverts significant scientific resources from real cutting–edge research and limits the possibility of European collaboration and coordination of clinical research. Clinical research funded mainly by marketing departments may have undesirable side effects such as impoverishment of standards of scientific excellence and steadily fewer incentives for the creation of networks able to deliver high–quality science.
7. Many countries in Europe have no defined training for clinical researchers. Only a handful of single institutes have combined training programmes in research and clinical practice (MD/PhD programmes) in oncology, and there are no pan–European initiatives. This lack of integration of translational research explains why although Europe has a strong foundation in basic science, we are still lagging behind the USA when it comes to transforming discoveries into new cancer prevention methods or into diagnostic tools and treatments available to patients. The United States has taken several initiatives to enhance its national translational research programme, including a recommendation by the 2004 Presidential Cancer Research Panel for more research programmes and significant funding to be devoted to translational research. The US–NCI has made funds available to build integrated translational research units in the major comprehensive cancer centres (CCCs), thus building a critical mass by encouraging cross–collaboration over state lines among cancer clinics and research units.

#### 6.2.3. Need for harmonization of methods in translational research

Efficient research requires access to large amounts of specific biological materials; biobanking is the science of storing tumour and tissue samples as well as biofluids. Europe has a good potential for biobanking, but unfortunately there are no common standards for collecting, freezing and storing tumours and biofluids, which is vital in order to be able to compare samples. Harmonization of standards and information files related to bio–samples must be implemented in storage centres to fully use the potential of existing facilities.

#### 6.2.4. Consequences of lack of integration and harmonization of translational research

Today in most European countries, there is little or no connection between research into cancer, which is usually performed in universities, and the practical treatment of patients, which is carried out in hospitals. There is certainly no such collaborative programme at the European level. Certain local exceptions exist, of course, some individual cancer centres are integrating research and care, but it is not enough, since these centres treat only a fraction of all cancer patients. As a result, new research findings are not becoming part of standard clinical care for regular patients.

#### 6.2.5. Conclusions

Thus there is a need for an overall vision for the entire translational continuum, starting with basic research and moving through early and late translational research into care and outcome research. Financial support of translational research activities by funding agencies at national level needs to be encouraged.

### 6.3. Lack of integration of epidemiological research with other types of research

The Europe Against Cancer Programme of the European Commission was a successful programme that launched an ambitious initiative aimed at reducing cancer mortality by 15% by the year 2000. The programme of activities and research focused on three major themes: Primary Prevention (particularly tobacco control); Secondary Prevention (screening) and Education and Training (also related to prevention). Moreover, the European Code Against Cancer was developed to foster health education and cancer prevention [17]. Without going into detailed figures, the Europe Against Cancer Programme appears to have been associated with the avoidance of more than 90,000 cancer deaths in the year 2000, a reduction of nearly 10% [18]. Unfortunately, this programme was cancelled.

Cancer incidence and mortality differ (sometimes considerably) between European countries, suggesting that a substantial percentage of cancers are avoidable. The prevalence of known cancer risk factors (such as smoking habits, alcohol consumption or obesity) varies across European Member States. We lack, however, a clear and uniform way of recording most of these risk factors in Europe, creating further barriers to meaningful research at the European level. Moreover, significant heterogeneity in the implementation of cancer prevention strategies exists between Member States, and there is little coordination of prevention research in the EU and within many Member States.

Institutions involved in epidemiology and public health work in different cultural and political environments have different research cultures and use different approaches. Training of health professionals in epidemiology and public health is highly variable across European countries. In a number of Member States, epidemiology and cancer prevention do not receive much attention in the clinical training and practice portfolio of health professionals, leading many clinicians and basic researchers to consider these aspects of the fight against cancer as ‘soft’ science. Most research efforts and research support is concentrated in the Nordic countries, the UK and the Netherlands, where debates on public health and healthcare tend to be more evidence–based in comparison with other EU countries. However, the professionals from these Northern countries rarely work abroad, for practical reasons as well as because the best opportunities for them are at home. Formulas should be found to facilitate the adoption of these practices in other Member States.

### 6.4. Legal and administrative barriers to translational research and to epidemiological research and surveillance

In the last decade, a number of new regulations, directives and corresponding guidelines have been issued, and a lot more will be following in the near future [19]. Consequently, the regulatory environment is becoming more and more stringent, and a paradoxical situation has emerged: on one hand, there is considerable demand from the public for effective therapies for cancer and better knowledge of environmental hazards, but on the other hand, concerns about confidentiality, and the protection of patients, healthcare workers and animals are leading to increasing numbers of legal and administrative barriers preventing or slowing down research. In particular, the EU Directive on Implementing Good Clinical Practice [20] has dramatically affected the regulation, oversight and practice of clinical research in Europe. The intention of the Directive was to protect patients and make the European pharmaceutical industry more competitive by ensuring that all Member States had the same rules. The weight of the directive at least doubled the costs of clinical trials [21], and increased the administrative burden so much that many academic researchers could no longer perform such trials. Consequently, the directive reduced the number of investigator–driven university–based trials. Transposition of the Directive into the legislature of Member States has led States to make additional changes, with often–tougher requirements on trial designs. The variety of interpretations of the Directive by Member States has led to many countries having different rules, hence increasing rather than decreasing disharmonies between EU Member States. For instance, the term ‘clinical trial’ normally should apply to ‘interventions’ in humans (with or without randomisation) done in the clinical setting. However, transpositions and interpretations have often been applied to any type of study (interventional and non–interventional, experimental or non–experimental) being done in clinical settings or outside hospitals. As a consequence, Sweden, for instance has seen a 25% decrease in academic clinical trials, Ireland 60% and Poland a staggering 90% reduction [22]. Trials that are conducted are usually delayed, to the detriment of both researchers and patient organizations.

Many databases with different medically relevant information on the same individuals do not communicate, often because legal barriers make the database owners unwilling to do so. Concerns about confidentiality have been unnecessarily exaggerated and have been fuelled by the passing of the European Directive on Personal Data [23] and by the Directive on Implementing Good Clinical Practices [24]. The intention of the Personal Data Directive was to harmonize the different policies on personal data protection existing in the EU Member States. However, this Directive and the variability of interpretations made by Member States caused considerable confusion [25]. For instance, in some countries, the legal status of cancer registries and data collection for these registries became problematic. In other countries, interpretations of the Personal Data Directive going far beyond the provision of the text adopted by the European Parliament, resulting in Kafkaesque situations [26].

In 2006, the UK Academy of Medical Sciences came to the conclusion that over regulation and overly cautious interpretation of regulation is stifling important research [27].

Misconceptions and legal and administrative barriers have multiple damaging effects, and not only on research activities. For instance, epidemiological surveillance is also impeded, when one of its important remits is the identification of likely causes of increasing cancer incidence and/or mortality because of linkages between large databases on cancer incidence, cancer mortality, risk factors and clinical data. These barriers weaken the little collaboration in many countries between the clinical and the public health communities and lack of access to many potentially useful data. This has counterproductive effects on scientific work and also on individuals who could benefit from added knowledge and discoveries that crosstalk between databases could permit. For instance:

Health threats posed by environmental agents may be left unrecognized because of the impossibility of linking the data relevant for uncovering cause–effect relationships between exposures of populations to these agents and cancer (and also other diseases).
Many biobanks created by clinicians do not have long–term follow–up of patients because of an absence of links with cancer registries, which are managed by public administrations.
The net impact of screening or treatments on cancer mortality is still nearly impossible to assess in spite of the wide availability of data from cancer registries and on treatments.
Many epidemiological studies cannot record data on relevant exposures such as drugs taken or medical examinations undergone. Such data linked with other data collected during the study would be of interest for pharmaco–vigilance purposes and to explore unforeseen beneficial or unfavourable effects of drugs.
The scientific community is also concerned by the many propositions that aim to reduce or even prohibit the use of laboratory animals in medical research. The community recognizes the need for ethical review of research that involves the use of animals, but the accumulating legislation surrounding animal experiments in medical science means serious delays in testing new cancer therapies in animal models.
Overzealous concern for animal welfare in the laboratory environment has led to bizarre consequences: in Sweden [28], it is prohibited to give a mouse an intra–muscular vaccine without a special permit from the agricultural department, something not required for the routine tetanus shot given to all newborn children.
The Commission should evaluate the direct, indirect and combined effects of the accumulation of Directives and of their transposition in Member States legal texts. This evaluation work has started for the Directive 2001/20/EC on clinical trials, and the research community would welcome this Directive to be amended as soon as possible with a ‘salvage paragraph’ to ease the restraints on academic trials [29]. Such amendment would greatly lower the burden on innovation in cancer therapies. Then in a second step, creating a legal framework that is truly common to the Member States for clinical trials would give an added value to conducting trials at home. Similar evaluation process should be initiated for other Directives related to personal data and life sciences.

### 6.5. Lack of functional contact with industry

The complex pattern of European research is a challenge not only to the research community itself, but also to biomedical and health technology companies. The lack of a coherent voice in cancer research and the many small academic research groups competing with each other makes it difficult for the industry to find world–class academic research bodies with sufficient critical mass and administrative structures able to create a real partnership with the industry, including running effective negotiation and managing intellectual property issues. The United States and certain Asian countries have spent years managing large–scale platforms with both the intellectual capacity and critical mass for research collaboration.

Conducting clinical trials in Europe has become increasingly expensive and difficult in recent years. As a result, multinational pharmaceutical companies are moving early trials away from Europe, and the remaining pharmaceutical trials that are carried out in Europe are increasingly being done for marketing purposes. As a result, the connections that are made today between academia and industry are more and more frequently initiated by the marketing departments of the pharmaceutical companies instead of their R&D departments. This is a most unfortunate development, as the scientific level for marketing trials is well below that of phase I, II and III trials, which is leading to an intellectual impoverishment of European clinical researchers.

The pharmaceutical industry, aware of the problems specific to the European research area, has launched the Innovative Medicine Initiative (IMI) whose first goal is the fostering of joint strategies between pharmaceutical research facilities and platforms of academic institutions to increase the efficiency of pharmaceutical research. In addition, there is a political will to support joint partnerships between academia and industry in order to retain innovation in Europe, and financing of IMI projects will be shared between the pharmaceutical companies and the European Commission.

The pharmaceutical industry is especially interested in the establishment of standards for biomarkers and alternative clinical endpoints, and would therefore like to access the biobanks, know–how and units for translational and clinical trials that are present in the cancer centres. Another underdeveloped area in the pharmaceutical industry is research in clinical trial methodology. The Innovative Medicines Initiative provides a perspective for fruitful collaboration with academia, but the absence of a sound European platform for translational research shows that much remains to be done.

### 6.6. Lack of functional information flow

Cancer research is, like any other discipline, embedded into the information age. Cancer connects a multitude of basic, clinical, epidemiological and public health disciplines. Due to the number of research areas, it is of vital importance that people have easy and speedy access to accurate and up–to–date information. Decision makers, journalists, patients, teachers and the general public expect simplified but accurate information on recent research developments.

As of today, there is no overall vision or structure on how to facilitate the flow of information on an European level [30]. It is impossible to find such simple information as who does what in cancer research in Europe or where and what technology platforms exist in basic or applied cancer science projects throughout the European Universities and Cancer Institutes [31]. There is no comprehensive database of open and ongoing clinical trials in Europe accessible to patients and doctors.

Cancer centres have a hard time marketing themselves and recruiting internationally, since most positions are only advertised locally and in the Member States official language(s). Certain types of information, such as grants and clinical guidelines are restricted to a linguistic and geographical area and are thus, in practice, inaccessible outside of the specific Member State.

The National Cancer Institute in Bethesda, MD, USA has a web portal to address most of these issues (www.cancer.gov). Some European countries, agencies or projects have developed useful web databases for their own needs, but there is no access point equivalent to the US–NCI web portal that answers to specific needs at European level.

### 6.7. Lack of mobility in research

The multitude of research institutes and programmes is the greatest strength in preserving variety in European cancer research. Regrettably, it is also one of its greatest weaknesses. Researchers and clinicians who remain at their alma mater all their working lives will be at a disadvantage when it comes to incorporating new ideas.

The lack of coordination in research training is striking. As previously mentioned, there is no joint PhD/MD programme on an European level in any of the cancer–related disciplines, which contributes to the lack of mobility for young researchers interested in translational research. Freedom of movement for people is one of the four freedoms guaranteed by EU law. There are several policy documents on the European level available promoting and enhancing mobility of researchers [32]. Nevertheless, an Eurostat report on the mobility of science and technology workers found that an average 5.7% of human resources in research and technology of the EU–27 are citizens of a foreign country, half of whom are citizens from other member states. The statistics, published in June 2007, show that there are large disparities in the share of these highly skilled people among EU countries [33]. Basic researchers willing to go abroad usually do a few years of post–doctoral work at another institution early in their careers to gain insight in a new methodology and be trained in novel patterns of thought. Yet there is an imbalance in how these exchanges occur. Many researchers prefer working in the USA or United Kingdom, which is partially reflected in how the Marie Curie grants are used. An Impact Assessment Analysis of the Marie Curie Actions of the 4th and 5th Framework Programme showed that the United Kingdom was the most popular host country (28% of all fellows), followed by France (17%), Germany (12%), the Netherlands (9%) and Spain and Italy (6% each) [34]. In the same analysis, the authors stated that (a) the Marie Curie fellowships seem to be a very useful and successful scheme, greatly appreciated by the majority of those who participated, both fellows and supervisors, (b) evaluation procedures were perceived as meritocratic and objective and (c) the quality of supervision was rated highly with the majority of fellows. The qualitative component of the impact assessment furthermore showed a high training impact of the Marie Curie fellowship, in terms of additional scientific skills, complementary skills and interdisciplinary experience. For many of the post–doctoral fellows, the Marie Curie fellowship allowed them to reach scientific maturity and gain independence as a researcher.

Many scientists who move to the USA do not return, due to the lack of an attractive career structure similar to that which they can expect should they remain in the North America. An additional barrier to movement for scientists wishing to pursue a few years of work outside of their own countries is the lack of a general and flexible European pension system.

Despite the possibilities of fellowship programmes both on national and EU level, clinical researchers especially have an even bleaker chance of travelling abroad in order to learn new skills. The career path of a clinician in general does not favour work outside his/her own institution. Furthermore, there are few short–term travel grants for clinicians similar to the EMBO short–term travel grants for basic researchers. Coordination of curricula for training of MSc, MD and PhD degrees and specialisation for the clinical fields is weak, impeding translational initiatives in education.

Our own data in analysing the life sciences–relevant Marie Curie fellowships of the 6th Framework Programme also show a relatively good coverage of basic researchers but almost no clinician mobility in oncology in Europe.

### 6.8. Lack of efficient funding

Funding in cancer research is a delicate question not easily answered. Existing funding structures are by and large national, through government–sponsored programmes or through charities. Only 5% of research funding comes directly from the EU.

Fragmentation of research is furthered by the fragmentation of funding agencies that usually give the preference to short–term grants of usually small amounts, to the detriment of long–term support to research programmes that need sufficient time for obtaining sound results. Most funds are obtained from national institutions that do not accept their funds being used for research carried out in other countries, and consequently, decrease the potential for transnational collaborations, and the possibility of national research teams acquiring new knowledge through international co–operation.

Grants are usually slow to obtain, with more than a year of planning required, and short–running, usually only two or three years, which presses the scientist to pursue low–risk projects with equally low innovation value. Many funding organizations do not evaluate the results of the research that they sponsor according to well–defined criteria commonly accepted by the scientific community as being indicators of scientific excellence [35].

Many researchers and research institutions notice that there is often a gap between the research avenues researchers want to explore and the possibilities set by funding mechanisms established by funding bodies and administrations; indeed, this problem is not limited to research in the cancer domain.

Researchers usually have grants from a few of these funding institutions. Scientists, by and large, are not in favour of decreasing the importance of national sources in exchange for a single European grant–giving body. This reluctance for greater EU funding at the expense of national funding is essentially due to fear of the bureaucratic burden imposed by the EU, and the lack of transparency of the peer–review processes, even though national funders may be less transparent than the EU. One of the most important reasons why so few clinicians are involved in research is due to the unwillingness of the social security systems in Member States to allow funds, infrastructures and work time for research purposes.

Academics are locked in institutional systems, and there they must spend more and more time writing applications and performing administrative tasks, which reduces the time they can spend on performing actual research. Translational research and research in quality of life for patients are particularly under funded. There is a need to fill the gap in funding between basic and clinical research to speed the transformation of new discoveries into clinical practice. The grants should have a long–term nature and should accommodate the flexibility required when working with patient’s materials. It is not the intention of this Project to suggest any large changes in funding mechanisms, which have evolved over many years. However, there is a need for a correction in the system so that it can swiftly fund emerging research and infrastructures on an European level and work over national borders when needed.

### 6.9. Lack of efficient networking

Networking has been often seen as a way to create ‘virtual research communities’ (or ‘virtual institutions’) [36].

The apparently obvious advantages of network building reside in flexibility, convenient ways to share resources, lower costs than those of real institutions and less administrative burden. The 6th Framework programme had the intention of creating networks as a part of its strategy for scientific collaboration. Funding networks through the 6th Framework programme (1 Jan. 2003 – 31 Dec. 2007) were intended to bring researchers from different Member States together. Reviews of the sixth component on Network of Excellence identified a number of aspects that needed improvement and simplification [37].

The promotion of networks has been particularly welcomed by academic institutions that would not have started trying to form networks if this instrument had not existed. However, many scientists, who were unused to or lacking in the human resources necessary to deal with the administrative requirements of the EU, considered the system difficult.

In the cancer area, except for some notable exceptions (see Chapter 5), coordination initiatives based on a ‘network only’ approach have proved unsustainable, and their influence on our ability to perform world–class translational, clinical and epidemiological cancer research has remained limited. The degree of complexity of much cancer research implies the existence of (a) a minimal management structure, (b) a long–term strategic vision, (c) long–term sustainability, and (d) the existence of minimal rules for institutions deemed to become part of a network.

## 7. Six agreed-upon solutions to surmount barriers to coordination

The Project underlines that much effort is needed to fill the gap between getting the actual financial investments in research to comply with goals set by the Lisbon Agenda, and for matching efforts done in the USA and increasingly in Asian countries. This being clearly stated, the Project concluded that the fundamental issue for cancer research in Europe is the lack of coordination and lack of support to truly innovative research areas.

Solutions outlined in this chapter are those identified during the Project as being most critical for overcoming the barriers depicted in Chapter 6. These solutions in collaboration with other successful biomedical initiatives will contribute to encouraging innovation and coordinating cancer research on the European level.

### 7.1. Proactive management of innovation and facilitation of collaborations while maintaining an atmosphere of healthy competition within the European cancer community

Although there is research in Europe of exceptional quality, it is not homogeneous in nature and is fragmented throughout the continent.

Proactive management would allow the launch of initiatives in areas that need special or rapid attention and allow the promotion of innovative research avenues. This would present an opportunity for stakeholders to reach a consensus and discuss topics in need of clarification as well as being a body capable of finding experts able to advise European institutions when needed. At the same time, proactive management could take care of maintaining healthy competition and avoid the advent of oligopoly in specific research domains with the risk of drying out creation and innovation.

Before launching expensive translational studies it would be useful to discuss with regulatory bodies issues likely to affect the design, conduct and analysis of translational studies. For instance, most potential anti–cancer agents must first be tested in phase I trials involving patients with advanced (metastatic) cancer. However, it is increasingly believed that some agents would be more efficient in patients with localized cancer. This constraint means that a phase I trial might not recognize the value of an agent of great usefulness in patients with early cancer. If we desire to screen agents for their potential anti–cancer effects in humans, the way forward is the use of biomarkers and of surrogate endpoints able to inform us on the likely efficacy of the agent, on individual and disease characteristics correlated with response or non–response and on side effects in patients with early cancer. Proactive discussions with regulatory bodies [i.e. the European Medicine Agency (EMEA) and national authorities] might define biological endpoints, valid designs and biostatistical aspect of studies.

Proactive management means functioning as a think tank, organizing workshops in fields in need of revitalization, proposing seed money to worthy projects resulting from these workshops and ensuring a fair and balanced review of the outcomes of the research. This will energise the field and encourage more competition between research groups. Taking the NCRI in the United Kingdom as an example (see Chapter 7), we could eliminate redundancy in research and help to direct the spotlight on underdeveloped research areas and malignancies.

Many funding organizations have no system for quality control of the research they support. Consequently, there is a need to monitor the impact of funding on research quality. Most clinical trials are run by industry for industry needs. Certain types of clinical cancer research, not usually supported by the industry, such as chemoprevention trials or early trials on biotherapies, deserve special attention. To preserve independent clinical research, it is desirable to implement strategies for further strengthening the coordination of academic clinical trials, such as the European Organisation for Research and Treatment of Cancer (EORTC) [38] or the TransBIG network for trials of individualised treatment of breast cancer [39]. In addition, as already mentioned, the Directive 2001/20/EC on clinical trials should be amended to keep clinical research alive and independent from the industry.

Cancers specific to an organ are now known to encompass different cancer subtypes with different responses to treatment and outcome. To understand why a given individual develops a specific tumour, epidemiological investigations (population–based or clinical–based) require very large sample sizes. Epidemiological investigation is also critical for the usefulness of biobanks and for understanding all the information provided by the ‘omics’ sciences: understanding the reasons of the occurrence and death from a cancer having defined molecular characteristics and dissemination potential can only be done in the light of genetic background, data on past exposures to risk factors, clinical data, data on treatments and long–term follow–up data.

Europe must continue taking advantage of its diverse populations and lifestyles to investigate the conditions needed for cancer occurrence and death. In this logic, epidemiological research should more and more be conducted across nations, as unveiling interactions between genetic background or metabolism features and exogenous risk factors will require enormous population samples.

Better communication and collaboration between basic researchers, clinicians and epidemiologists, both within and between Member States, is needed to advance science in several fields, including that of gene–environment interaction, where very large studies and multiple expertises are essential.

#### Likely consequences of not having proactive management

Cancer research in Europe does not exist in a vacuum; it is continually interchanging with other continents. Without proactive management to encourage and coordinate contacts between basic and epidemiological research centres, clinical settings, public health offices, regulatory bodies and similar structures abroad (in the USA, China or India, e.g.), the global view will be lost and the European cancer research community will have lost precious time for taking decisive steps, thereby slowing down the translation of basic and epidemiological research into clinical and public health practice.

### 7.2. Establishment of an information exchange portal (brokerage of knowledge) for health professionals, patients and policy makers

The lack of efficient communication (including fast dissemination of results) among stakeholders is a major barrier to collaboration in Europe. The sheer vastness of available information makes it impossible for any single individual to have a complete and detailed overview of the field. The load of information, coupled with it not being presented suitably for its target audience, leads to sub–optimal use of intelligence and slows down the innovation process as well as the impact of research findings on health.

Many web portals on cancer science exist in Europe, often designed for local or national needs and not providing services covering all Europe, such as:

List of epidemiological studies, clinical trials and translational research studies proposed, in progress and finished (with results).
List of medical facilities and research centres with their specific research domains and networks.
List of medical facilities having the human resources, the technology and links with other facilities (see platforms in next section) dedicated to translational research.
Lists of institutions offering research grants and searchable databases for funding opportunities.
Guidelines for clinical trials, translational research, epidemiological studies and other types of applied studies.
Recent development in treatments.
Lists of national cancer plans.
Lists of scientific meetings.
Portals for human resources (jobs offered).

We must create and maintain a complete and comprehensive web portal with multiple access points for scientists, healthcare professionals, patients and the general public to help them navigate through these oceans of information. The organizing structure should not store this data itself but provide a sensible and structured way to access it as well as disseminating relevant and new information on recent discoveries that may interest patients and the general public. Complete searchable web–based databases must be created on clinical trials, and other types of clinical studies being conducted in Europe with explanations as to why a particular study is being performed. Such databases will also present trial findings in a way directly usable by health professionals and patients. Scientists will have an European–wide database on grants and upcoming meetings as well as access points to databases.

The cancer research community must set up and manage, in collaboration with existing expertise at the International Agency for Research on Cancer (IARC), an epidemiological database equal to the Surveillance, Epidemiology and End Results (SEER) [40] in the USA [41].

#### Likely consequences of not building the information exchange portal

Should Europe fail to adapt to the continuously increasing volumes and sophistication of the scientific information in the cancer research field there will be numerous consequences, starting with the intellectual impoverishment of the European research community. Without a functional exchange system for accurate information, the present–day confusion and disarray will continue and grow, which will result in lost opportunities to forge connections that might otherwise spark ideas for innovation and interdisciplinary collaboration across our continent.

Access to information will be restricted for patients who may not know how to find it. Physicians treating patients will not have access to clinical trials. Results from trials and from epidemiological studies will be confined to specialists and will not reach their potential beneficiaries and decision makers. Scientists will be limited in their ability to quickly access and survey information not directly linked to their own subspecialty projects. Treatment and cancer plans will be sub–optimal due to a lack of comparison with other European States. Access to important resources will be impeded, slowing the innovation process. Misinformation, such as food scares, will continue to flourish in the media.

### 7.3. The provision of guidance for translational and clinical research including the establishment of a translational research platform involving comprehensive cancer centres and cancer research centres

Management of cancer patients is increasingly multidisciplinary. Numerous studies show that quality of care is better in cancer centres that (a) have high patient load and have thus the necessary experience for provision of highly specialized care, (b) organize multidisciplinary care involving oncologists, surgeons, radiotherapists, psycho–oncologists, rehabilitation services, etc and (c) have significant research activities. Hence, multidisciplinary approaches to cancer also involve basic and applied research, as well as epidemiology and community health.

#### 7.3.1. The need to attain a critical mass

Future cancer research must innovate by identifying new targets for prevention, diagnosis and for the development of new treatments. As mentioned above, world–class research can best be achieved when a scientific critical mass is attained capable of performing the extensive, long–term research on new technologies or treatment modalities from basic research to delivery to patients [42].

Critical mass also encompasses:

Access to sufficient numbers of patients having specific types of cancer (including rare cancers or rarer subtypes of cancers).
The existence of administrative structures able to create real partnerships with industry, including effective negotiation and management of intellectual property issues.
Coordination, so that common views serve as guidelines for the development of research programmes.
Harmonization of technical processes in order to increase research quality.
Harmonization of administrative processes in order to decrease the bureaucratic burden and speed–up research meriting rapid attention.

Several countries have succeeded in (USA, Canada) or are in the process of (India, China) building large cancer centres gathering sufficient critical mass. In Europe, it is very unlikely to have similar huge medical structures providing high–quality care to cancer patients coming from all over Europe, due, among other things, to language differences and minimal cooperation between national social security institutions.

The United States has a long–term commitment to integrate Comprehensive and Basic Cancer Centres with excellent results. Europe has the potential to develop some world–class infrastructures for cancer research. Population–based patient materials are available for clinical trials. Biobanks and patient data registries can cover total populations of patients in defined geographic areas permitting the development of clinical and molecular epidemiology of high class. So, in Europe, a necessary step to overcome the present fragmentation in cancer research is to establish a translational research platform among the CCC, the basic and the epidemiological research centres in order to create the critical mass necessary to accelerate and expand the existing research.

#### 7.3.2. Comprehensive cancer centres

The CCCs are clinical centres committed to care but also research, prevention, education and innovation, and they need to interact with the relevant healthcare system. The concept of a CCC is a consequence of the globally increasing cancer burden, the complexity of cancer activities and the need to innovate in cancer care and prevention. To date, the most successful CCCs are those in the USA with the US–NCI as coordinator. The US–NCI has defined a set of criteria on comprehensiveness, the main themes of which are quoted below [43]:

A comprehensive cancer centre has reasonable depth and breadth of research activities in each of three major areas: laboratory, clinical, and population-based research, with substantial transdisciplinary research that bridges these scientific areas. A comprehensive cancer centre is expected to initiate and conduct early phase, innovative clinical trials and to participate in the NCI’s cooperative groups by providing leadership and accruing patients to trials. An NCI-designated Comprehensive Cancer Centre must also demonstrate professional and public education and dissemination of clinical and public health advances into the community it serves.

##### Major research areas of a centre

Multidisciplinary and transdisciplinary interactions between basic, clinical and prevention, control and population research: A cancer centre should feature vigorous interactions across its research areas and facilitate collaboration between basic, behavioural, epidemiologic and clinical scientists and between laboratory, clinical and population science programmes. These collaborations should facilitate rapid transfer of clinical observations to laboratory experiments and promising discoveries in the laboratory to innovative behavioural and medical applications in prevention, detection, diagnosis, treatment and survivorship. In geographic areas with multiple cancer centres, collaborations among centres may be appropriate. Centres having only basic research components are encouraged to seek collaborations with clinical units elsewhere, with industry, and with the NCI to facilitate the translation of fundamental discoveries into tangible patient benefit.

No particular organizational configuration is mandated by these guidelines. The organization should serve the science and be appropriate for the institution. The centre should have reasonable breadth and depth of scientific faculty, as well as adequate and appropriate facilities dedicated to each of the centres research areas.

*Basic research:* Centres should use their base of support to promote multidisciplinary interactions between scientists engaged in basic cancer research and, where possible, to stimulate collaborations among investigators in basic research and other areas.
*Clinical research:* A cancer centre should be a major source of innovative clinical studies that can be exported, e.g. to NCI’s co–operative groups or directly into general medical practice. Clinical studies should involve relevant laboratory research whenever possible. In addition to fostering translation between laboratory and clinic and conducting early proof–of–principle clinical trials, cancer centres should participate in NCI’s clinical co–operative group trials.
*Prevention, control and population research:* Cancer control research is the conduct of basic and applied research in the behavioural, social and population sciences that, either independently or in combination with biomedical approaches, reduces cancer risk, incidence, morbidity and mortality. The scope of this research is extensive, ranging from pre–intervention behavioural and bio–behavioural research, randomised clinical trials involving healthy populations or survivors, to research focusing on dissemination and diffusion of effective medical and behavioural therapies. Prevention research is directed at healthy populations, including those at high risk and/or those with detectable pre–cancerous lesions and cancer survivors. Not every cancer centre will conduct research in all aspects of prevention, control and population sciences. However, centres should demonstrate grant support not only in epidemiology, but also in several other areas of primary prevention, early detection, health services, dissemination, palliation and survivorship research.
*Interactions with Industry:* Cancer centres may serve as important interdisciplinary research platforms for evaluating the most promising industry products for early detection, prevention, diagnosis and treatment of cancer. Centres are therefore encouraged to engage in scientifically promising studies with industry, of new diagnostic tests or equipment and therapeutic agents, through clinical trials designed by the centre as well as field–testing new technologies important in the discovery process. Such studies can benefit from Cancer Centre Support Grant resources if the centre plays a key role in both the intellectual and operational aspects of the study, findings are made available to the biomedical research community and the study is consistent with current federal regulations regarding use of grant funds involving industrial partners.

In addition, more specifically for Europe, CCCs represent the optimal conduit for speedy uptake and evaluation of ‘improved’ developments from cancer research into routine care in the surrounding geographical areas, e.g. in local and regional healthcare institutions.

#### 7.3.3. Platform for translational research

A platform for translational research is a formal collaboration between a number of CCCs and institutions active in basic and epidemiological research for the creation of the critical mass necessary for conducting world–class translational, pre–clinical and clinical cancer research as well as the validation of prognostic and diagnostic markers. Such a platform would represent a key element for creating a culture of discovery, scientific excellence, transdisciplinary research and collaboration. With modern information technologies effective collaboration can be extended and become more systematic to meet the needs of globalization.

The creation of a platform will start a process of harmonizing the centres’ infrastructures, quality procedures, quality control of assays, standardization of biobanking procedures, etc. In particular, this platform should disseminate a harmonized culture of quality, for both research activities and medical care.

Hence, creation of a platform will require the pooling of significant volumes of resources (including entire laboratories, biobanking facilities, technical platforms, and exchanges of scientists and of expertise) and harmonization of patient’s files, of electronic data organization, of biobanking processes, and of laboratory procedures.

Membership in the platform should be open, and centres selected according to well–defined criteria and with outside help (e.g. peer review of applications for becoming a CCC by American and Asian experts).

One issue at European level is the differences in developmental stage and available resources of centres in the different countries, especially between the EU–15 and the New Member States. Less well–developed centres could, for example each be twinned with a more experienced one for mentoring.

A platform for translational cancer research will stimulate further collaboration from across Europe and the world. There are already several examples of such collaborations between European organizations and US or Asian groups.

#### 7.3.4. Platform for translational research and research infrastructures

European scientists and healthcare professionals need access to research infrastructures such as technical platforms, bioinformatics, biobanks and patient registries. Creation of a translational research platform will enable sharing of costly high–tech research infrastructure between different CCCs or research centres. This form of collaboration will require the development of true partnerships.

Cancer researches should also be more involved in the European initiatives deemed to create a world–class research infrastructure in Europe, although funding of these infrastructures seems to remain problematic [44]. Responding to the European Council’s invitation, the European Commission has set up a high–level Expert Group with representatives from all Member States that issued a report in February 2002, recommending the creation of an European Strategy Forum on Research Infrastructures (see Chapter 11.4). It is important that these programmes develop so as to take into account the special requirements and the particular infrastructural needs of translational research in cancer.

#### 7.3.5. Platform for translational research and economic outputs

Economic partnerships, such as those that create SMEs, are mandatory to fully utilise and market innovations. Scientists, who are driven mainly by an interest in research, usually find it difficult to cope with the legal and financial requirements of building–up companies of unsure economic future.

There is need for better guidance among researchers about intellectual property rights and start–up solutions for small enterprises. There should be an European structure for helping scientific–industry partnerships proliferate, finding the right clinical, legal and financial contacts necessary to bringing innovation into fruitful projects, of benefit to a larger market. In this respect, creating long–term programmes through a platform of translational research will help ensure continuity among research networks.

#### 7.3.6. Preserving healthy competition

It is necessary to find the balance between collaboration and competition, as overcoming fragmentation requires collaboration yet securing innovation and quality necessitates competition.

Linking a number of CCCs and basic and epidemiological cancer research centres should not result in a rigid structure incapable of change. Activities and scientific productions must be evaluated, meaning that over time the network must be flexible, welcoming new participants yet able to exclude partners not fulfilling their roles.

Research groups will thus not be inhibited in their competition with each other. Even if the CCCs harbour basic and pre–clinical research, most basic research projects will come from other research centres. Centres and units who are especially interested in a certain area, may chose to deepen their collaboration, but they may well compete with other centres or units. Funding agencies will be responsible for maintaining competition between networks and evaluating the scientific and economic outputs of funded research.

##### Likely consequences of not having management by CCCs, in platforms and research networks

The lack of critical mass in many research areas, with absence of harmonization, will affect cost–effectiveness and iteration of research projects. Sub–optimal educational and exchange actives will ensure that research, researchers and ideas will continue to leave Europe. Many ideas will remain dormant on European lab benches due to lack of adequate counselling on how to pursue a strategy for clinical testing and commercialisation.

As a result, Europe’s competitive position in cancer research will be weakened and cancer prevention and management of cancer patients will depend on scientific agendas developed in North America and in Asia.

### 7.4. Coordination of calls and financial management for cancer research projects

Funding mechanisms in Europe are richly varied, reflecting the research they support. Though this system has led to prolific basic research, there is little to match it in translational and clinical research. These disparate research–funding systems foster fragmentation and duplication of research activities, making it difficult to support translational research projects that require several years to develop. This is especially true for the funding of costly research infrastructure, vital to successful innovation. The intrinsic slowness of any funding process makes it hard to respond rapidly to emerging situations. Outside of industry, funding for cancer research is geographically limited as it comes from Member States; therefore, clear benefits will come from the coordination of funding plans and having funders enter into formal discussions, something the UK–NCRI has demonstrated.

A grant–giving body combining healthy competition and a top–quality review process should manage funding on the European level. It should be administered with speed and flexibility with regards to the cutting edge research of today in a multitude of cancer–related disciplines. Such a grant–giving body should be led by the funders themselves, and such coordination between funding agencies is probably the best guarantee of more efficient coordination of cancer research in Europe.

A review of the US–NCI Research Project Grant (R01) model, the original and historically oldest grant mechanism used by the NIH in the USA, is recommended [45]. Funding should encompass not only research grants, but also support infrastructures needed for high–throughput analysis and for sophisticated but repetitive laboratory analyses. Harmonization of clinical practice guidelines is being investigated by the Coordination of Cancer Clinical Practice Guidelines Project (CoCanCPG). The infrastructure aspect is now partly tackled by the ESFRI Programme supported by the European Commission (see Chapter 11), but there is urgent need to clarify the collaboration between the ESFRI programme and cancer centres involved in translational research.

#### Likely consequences of not being able to administer funds

By not bringing funders together, there will be a large amount of duplication in the system, yet underprivileged areas of research will continue to be left neglected. Does Europe really need 20 national programmes on breast cancer, when there is none on pancreatic cancer, the fourth greatest cancer killer of both men and women?

### 7.5. The construction of a ‘one–stop shop’ as a contact interface for industry, small and medium enterprises, scientists and other stakeholders

The pharmaceutical industry is constantly looking for easy, fast and inexpensive ways of doing clinical research, especially bringing laboratory findings into (fast) clinical testing to identify the research directions most likely to be successful.

More phase III trials (i.e. trials needed for registration) are being conducted in developing countries because of the rising costs and delays in Europe. Yet the pharmaceutical companies are interested in the high academic excellence and the clinical research potential that exists in Europe, especially for phase I (setting of dose range and side effects) and II (type of target cancer and biological/clinical response to treatment) trials.

There is a pressing need to make Europe more attractive for clinical research. Although trials for marketing purposes are still comparatively strong in Europe, we need to facilitate the performance of early phase proof–of–principle trials. The pharmaceutical industry and the EC have, through the IMI), created a platform to address this problem (see Chapter 11). The IMI has already requested that the Project helps create an academic partnership in cancer research to address mutual questions.

The creation of a one–stop shop will allow pharmaceutical and health technology companies to liaise with a platform of cancer research centres and to utilise their infrastructure and know how for biologically driven, translational research projects. Through the one–stop shop companies would also have access to the vast databases of patients’ registries and tumour banks that are present in academic research. Such collaboration would especially enhance research in rare malignancies.

Industry also wants clear and relevant guidance in the development of clinical methodology and will look to academia in universities and CCCs for this type of information. The industry also needs a third party to talk with regulatory bodies (EMEA and national regulations agencies), patients’ organizations and academics in order to discuss more general issues. The one–stop shop may serve as a portal for such connection as well as being a disseminating point between industry, academia and healthcare professionals.

Small and medium enterprises have different problems than large pharmaceutical companies, as they are usually restricted geographically and have only a small number of projects in their pipeline. The one–stop shop function would allow SMEs to connect to relevant partners, for instance to carry out early toxicological and first–in–man studies. The one–stop shop should also help to remove the current major bottleneck of identifying, which clinical partners would be best suited for expanding the findings from basic research into a clinical phase.

#### Likely consequence of not forming a one–stop shop

By not creating a common interface between academic and pharmaceutical research, there is a risk that more R&D activities and clinical trials will be moved from Europe to Asia, Latin America or even the USA. The consequence will be a decline in industrial research in Europe a reduction in good quality academic research supported by the industry, loss of jobs and of taxes, as well as a deceleration in the introduction of new drugs in Europe.

### 7.6. Support for greater involvement of health professionals in translational research and multidisciplinary training

The creation of a pan–European platform to discuss educational, scientific and healthcare with stakeholders like government agencies, universities, hospitals and research institutes could have a profound impact in fostering a culture of comprehensiveness, leading to training many more MDs in research techniques. Cancer patient care is more and more organized in multidisciplinary teams, and applied research is under the responsibility of these multidisciplinary teams. In this respect, clinicians should be able to spend more time on research than they are able to today. To fully integrate cancer research with care, healthcare professionals or multidisciplinary teams should be able to devote part of their time to research, while still financially supported by the healthcare system. Today basic researchers are not well enough informed about important clinical questions, and similarly clinical researchers cannot follow the rapid developments in the basic research area. A functional translational research process requires that researchers and clinicians come together from many different fields, each bringing a unique viewpoint and expertise. Integrating basic and clinical researchers will help to create a culture of mutual understanding. By fostering a culture of mobility and dismantling the barriers between the research institutes and clinics, we should be able to fully utilise the potential that exists in Europe today.

Training and mobility should be combined in an integrated process. Improved education together with increased possibilities for exchange of researchers between centres will be a strategy to retain and attract young investigators in Europe but also to recruit internationally. Just as clinicians should be able to spend more time on research, they should also be able to go to other cancer centres to exchange innovative ideas for treatment and prevention. There should be an European body, which could manage funds for training and mobility in cancer research and care. Education is a natural part of research. We must create and harmonize MD/PhD curricula and extend possibilities for post–docs to enhance training and mobility in translational research too.

Most important is scientific education aimed at having clinicians acquire the methods necessary to develop coherent high–quality research processes. In this respect, the Project recommends the creation of European curricula (e.g. Master’s degree) in translational research open to health professionals and basic scientists.

#### Likely consequences of not supporting training needs

If we neglect education in cancer care and research today, we will face severe recruiting problems in the future. The translational research process will be dysfunctional, due to gaps where trained specialists are lacking. Failure to harmonize educational platforms will lead to discrepancies within the oncology field in Europe, rendering it more difficult to support free mobility among future researchers and clinicians. Ultimately, differences in training will lead to variable quality of cancer research.

## 8. Recommendations of the Eurocan+Plus Project

How a research community is framed can have dramatic effects on its productivity, focus and overall impact 46. The Project considered that currently available European legal instruments were not adequate for addressing issues on barriers depicted in Chapter 6 and implementing on the long–term solutions presented in Chapter 7.

There is a thus a need for an instrument viable in the long term and able to structure, facilitate and nurture ambitious innovative cancer research based on synergistic networks of basic, clinical and epidemiological researchers and their institutions. This structuring capacity would be crucial for fostering, among other things, a translational research culture. Therefore, it is the recommendation of the Project, adopted by the General Assembly of 19 November, having taken into consideration the consensus view of the barriers to collaboration and the agreed solutions and having examined the existing European structures (see Chapter 11), that:

1. A small, permanent, efficient and low–cost European Cancer Initiative should be formed. The goals of the ECI would be:
  - Become the common voice of the cancer research community and serve as an interface between the cancer research community and European citizens, patients’ organizations, European institutions, Member States, industry and SMEs.
  - Stimulate innovation in cancer research and facilitate research processes through putting into practice the solutions described in Chapter 7 aimed at alleviating barriers and coordinating cancer research activities in the EU.
  - Have the capacity to deal with legal and regulatory issues. The ECI should be proactive and duly represent the European cancer research community in processes leading to legal texts likely to affect research, like for instance the processes that resulted in the European Directive 2001/20/EC on Implementing Good Clinical Practice.
2. The creation of an ECI will be a complex process. As an initial step, coordination efforts should be directed towards the creation of a platform on translational research, which will imply:
  - Definition of the coordination between basic, clinical and epidemiological research in order to create the best scientific, technical and administrative environment for the undertaking of major translational research projects.
  - Formal agreements of cooperation between CCCs going beyond collaborations, that is forming durable partnerships by pooling significant volumes of resources (including entire laboratories, biobanking facilities, technical platforms, exchanges of scientists and of expertise) and harmonization of patient files, of electronic data organization, of biobanking processes, and of laboratory procedures.
  - Networking between funding bodies at the European level in order to allow joint initiatives for financial support of promising multinational translational research projects.
  Funding agencies will be responsible for maintaining competition between networks and evaluating the scientific and economic outputs of funded research.
  The translational research platform can be considered as the natural continuation of the Project. Through its constitution and processes it will put in place, the platform will address the essential political, administrative and technical steps necessary to establish an ECI.
  The initiation of a platform for translational research could be based on an ERA–NET approach, or newer versions of this type of instrument involving comprehensive cancer centres and funding bodies willing to provide adequate support to translational research activities. This ERANET will aim at examining types and modalities of collaboration between basic, clinical and epidemiological centres and how the platform should be structured for the sustainability of activities. This structuring of the platform will serve for founding the basic management tools of the ECI.
  The platform for translational research will be in part based on the development of a network of comprehensive cancer centres with formal agreements between these centres. Recourse to non–European experts for definition of criteria for labelling of CCCs is an idea positively considered by most health professionals working in oncology settings. The already existing networks (e.g. OECI, EACR, EORTC NOCI, the Italian Network of Cancer Centres) will be part of the definition of this project.
3. The constitution of an European database on cancer research projects and cancer research facilities should also deserve attention as such service is crucial for the coordination and management of research. Initiation of this service could be based on an ESFRI type of approach, and could capitalise on already existing experiences in Europe and the USA [47].

The Project examined other coordination modalities, some of which are of interest, but cannot be a substitute for an ECI.

Traditional European legal instruments and other EU agencies.

The way the ECI may function over the long term is quite different than technical structures like the EMEA or the EFSA. These institutions entail significant amounts of administrative management with recurring processes. Recent complex initiatives, like the Innovative Medicine Initiative (see Chapter 11) could not be mounted using traditional EU collaborative research instruments, as these instruments cannot achieve the coordination of research efforts necessary to cope with the scale and complexity of the research challenges involved. Therefore, the Joint Technology Initiative (JTI) was introduced in the Seventh Framework Programme (FP7) as a new way of realising public––private partnerships in research at the European level.

The Article 169 of the Maastricht Treaty covers joint funding initiatives. The only project developed under Article 169 was the European and Developing Countries Clinical Trials Partnership (EDCTP), and it has failed to fulfil its objectives [48]. Additionally, the administrative procedures required by Article 169 are so unwieldy as to make it unpractical for the current project.

There thus remains a need to define the legal environment that could shelter an idea like the ICI.

### Why not simply an ERA-NET of funding agencies?

A network of funding agencies would only represent a partial response to fragmentation issues. Most researchers would feel excluded from such a network. As a result, it would have little capacity to foster the critical mass needed for translational and other types of research. In addition, it leaves unaddressed a number of problems, like involving clinicians more in research.

### Why not simply adopt a bottom-up approach?

In essence, bottom–up approaches proceed from creativity and innovation. For cancer coordination in Europe, these approaches have worked well for specific areas of research, especially basic research. As a result, many research domain or disease specific networks and organizations exist in Europe. These are usually limited in terms of numbers of institutions involved and in their ambition. None of the bottom–up attempts in the last decade have succeeded in establishing a sustainable, functional network involving major CCCs in Europe; nor have they fully addressed the issue of fragmentation. There is thus a need for a (minimal) management structure that can orchestrate bottom–up initiatives, allowing them to work together, provide support for their sustainability and encourage occurrence.

### Why not create a network of (major) CCCs, for creation of a virtual ECI?

The Project discovered that in spite of much scientific collaboration, there has been no significant formal cooperation between major European cancer institutions going beyond classic collaboration with pooling of significant human and technical resources. Only recently has collaboration between cancer centres become recognized by larger numbers of institutions as a priority for the future of applied cancer research in Europe.

Comprehensive cancer centres, however, are not representative of the entire cancer research community and they cannot cover many aspects of cancer research (e.g. being a one stop shop or information portal, discussions with regulatory bodies). In view of past experience, a network of CCCs without central coordination is probably unrealistic, in terms of managing projects in the network, peer–review procedures for qualification of CCC, maintaining sustainability of the network, administrative requirements for funding and sharing of research infrastructures and attracting talented researchers.

The experience over the last decade has shown that ‘virtual research communities’ are rarely sustainable in the long term (at least in the cancer field) and depend much on the devotion of some scientists. When these scientists leave the scene, the virtual community disappears. Without being sustainable on the long run, no major achievement can be expected in complex endeavours as those needed for efficient translational research or long–term epidemiological research. Sustainability requires formal coordination of resources that have been put in common, with a mechanism ensuring continuous funding of that coordination (even if minimally expensive) and of exchanges between institutions involved in networks.

### Why not develop the ECI idea in an already existing institution?

In the current situation, it is unlikely that major cancer institutions would be eager to join or to merge with other institutions. The ECI has the merit of being a new structure, and for major cancer institutions it will be easier to join a new structure than to have the impression of becoming secondary to another already existing institution.

## 9. Proposal for an European Cancer Initiative (ECI)

The ECI model proposed in this chapter was inspired by the UK NCRI [49] and by the European Molecular Biology Organization (EMBO/EMBL) [50]. It is vital that the ECI gains legitimacy among all stakeholders, that is cancer researchers and research institutions, patients, the general population, funding bodies and governments. The ECI must therefore be a neutral, independent body managed by the stakeholders themselves, with the understanding that funders will represent the main decision making structure. The ECI should be self–sustainable, which requires stakeholders to show long–term commitment to the initiative.

### 9.1. How the ECI would work?

The ECI should act through:

1. Promotion of innovation, creativity and scientific curiosity.
2. The gathering of multidisciplinary expert groups for defining main directions for research and core research activities or infrastructures that should be created or further developed.
3. Promotion and structuring of coherent, sustainable research groups and networks allowing collaboration between experts in cancer research.
4. Facilitate availability and exchanges of information relevant to researchers, clinicians and patients.
5. Fostering healthy competition between research groups and networks of institutions.
6. Avoiding unnecessary bureaucracy and simplifying existing complexities whenever possible.
7. Training.

The core added value of an ECI lies in its capacity to manage the complexity of cancer research with maximal flexibility and minimal bureaucracy, and its ability to rapidly identify areas needing swift action. The ECI should tap into and optimise pre–existing EU resources and will therefore not perform its own basic, clinical or epidemiological research.

### 9.2. ECI functioning

Decision mechanisms in the ECI should adopt both bottom–up and top–down approaches [51]. The dosing of the bottom–up and top–down approach would be variable according to the issues at stake, expectations from the research community and the coordinating role of other institutions.

The proposed structure and working patterns of the ECI will have as an essential goal to ensure smooth circulation of ideas and initiatives from members of the research community to decision–makers (bottom–up) and reciprocally (top–down). For instance, the ECI should come to a consensus definition as to what constitutes an European CCC. Accreditation of medical centres applying for the CCC status should be done independently, with perhaps recourse to non–European experts, and this process will be essentially of a top–down nature. On the bottom–up side, the ECI will foster discussions among CCCs as to cooperation in terms of resource sharing, strategies, solutions to common problems, money for meetings, training programmes, exchange of MDs and scientists, etc.

### 9.3. ECI Organisation

The following figure summarizes the main features proposed for the ECI [52].

#### Basic structure proposed for the ECI

##### The Board of Funders (BOF)

The Board of Funders (BOF) should constitute the highest governing body of the ECI and has the ultimate responsibility for the organization.

The BOF should be open to the European institutions, in addition to governmental and non–governmental agencies funding quality cancer research established in EU countries. These institutions/agencies should meet the following criteria:

1. Devote a flexible amount of financial resources each year for cancer research in the EU.
2. Accept to contribute to the running budget of the ECI, on a pro rata basis, according to the budget each funder allocates to cancer research projects. The non–governmental agencies receiving funds from other bodies while being funders, and the research groups generating research incomes but also receiving funds should not be part of the BOF, but part of the External Stakeholder Assembly described below.

##### The Scientific Advisory Board (SAB)

The SAB should be constituted of experts in both the basic and applied cancer research fields. It is nominated by, and will interact with, the BOF as needed. The BOF should nominate a Chair of the SAB who will be in charge of organizing the activities of the SAB and oversee conflict–of–interest issues.

##### External Stakeholder Assembly (ESA)

The ECI will need the valued judgment of professional organizations in Europe to shape the optimal future for European cancer research. Typically, the ESA should include institutions or non–governmental organizations that are not ‘pure’ funders of cancer research.

The ESA should comprise organizations like the IARC, EORTC, OECI, EMBO, ECCO, patients’ organizations, individual cancer centres and public health associations. These organizations should be invited to give advice to the BOF. It shall be an open and inclusive assembly. Becoming a member of the ESA should be subject to approval by the BOF.

##### The Executive Office (EO)

The BOF should establish an executive office led by an appointed Director who will have a small professional staff at his/her disposal. The executive office should have as its principal duty to coordinate and manage the Standing Committees. The Director should be responsible for day–to–day management issues and should be held accountable to the BOF for the actions of the Office. The daily tasks should be performed by professional and dedicated staff employed by the ECI subject to approval by the Director. A key responsibility of the EO will be to facilitate transmitting bottom–up initiatives from researchers to BOF and other relevant institutions, and to organize smooth, efficient flow of information from BOF to the research community.

##### The Standing Committees (SC)

The executive office should form proactive SCc for taking care of the solutions described in Chapter 6.

##### Temporary Committees (TC)

Temporary Committees, to address specific issues, would be created on an ad–hoc basis.

## 10. Short-term plans

The Eurocan+ Project may pursue its activities in the following directions:

10.1. Disseminate reports and conclusions of the Eurocan+Plus Project

The results of Eurocan+Plus will be disseminated through contacts with MEPs (e.g. the MAC MEPS), the EC, the Member States, Academics, Research centres, etc.

Several articles will be prepared, beginning with one summarizing the main issues addressed by the project (objectives, process, barriers, functions, ECI).

10.2. Pursue efforts for establishing a platform for translational research [53]
10.3. Initiate discussions about the physical establishment of the ECI and of its functioning.
10.4. Work Package 11 will pursue discussions with the IMI project and define areas of collaboration.
10.5. Contribute to training activities alongside project proposed by the IMI and the EMBO.
10.6. Establish discussions with existing ESFRI programmes, especially EATRIS and ECRIN for further collaboration.
10.7. Become involved in the ESFRI Roadmap Working Group for Biomedical and Medical Science.
10.8. Support and promote ‘Fill the Gap’ project for equality in European health.
10.9. Support and promote CoCanCPG for research on guidelines for clinical practice in Europe.
10.10. Disseminate information on national cancer plans and promote an European framework for standards of national cancer plans.

## 11. Examples of European organizations active in cancer research

In our considerations, we found the following projects pertinent and worthy of mention because they represent examples of projects that have succeeded, with vary degrees of hardship, and can act as examples for Eurocan.

### 11.1. TuBaFrost (www.Tubafrost.org)

The TuBaFrost project illustrates the need for sustainability in cancer research. In 2002, the EORTC, with funding from the European Commission FP6, launched a virtual tumour bank project called TuBaFrost, to create a network of frozen human tumour tissue banks across Europe.

TuBaFrost is a consortium of 20 pathology and research departments based in hospitals involved in care of cancer patients and cancer research. The consortium developed an infrastructure to enable the linking of tumour tissue banks into a network with minimal impact on local tissue banks, by collecting parts of the tissue biopsy not used for diagnosis or kept for local archives and usually discarded.

TuBaFrost developed standards for frozen samples intended for translational research and solved the legal aspects concerning the exchange of tissue samples across European States. Tissues are frozen in a standardised way and all samples for the collection are anonymised. A secure TuBaFrost electronic database stores information about each tissue sample: type of cancer, type and quantity of tissue collected, collection and storage details, clinical outcome of the donor. The frozen tissue is stored in the hospital where the donor was diagnosed/treated and stays there until used or sent to another hospital or research centre within the TuBaFrost group. Tissue samples within the TuBaFrost collection will only be used for research approved by ethics committees. The patient has the right to withdraw consent for the storage or further use of left–over tissue at any time.

The electronic database tracks samples throughout the network. The exchange of tissue to other hospitals is regulated by a contract, which uses the national regulations of the country supplying the tissue. In addition, the TuBaFrost website will display links to published research papers resulting from the TuBaFrost project.

After several years of work devoted to the development of standards for tissue collection and procedures for transnational tissue exchange, the TuBaFrost project is ready to become fully operational at the very point when support from the European Commission has been discontinued. TuBaFrost is currently supported by the Organisation of European Cancer Institutes (OECI) and is threatened by termination if more funding cannot be made available.

### 11.2. National Cancer Research Institute—NCRI (www.ncri.org.uk)

Cancer research coordination within the UK has improved because of the National Cancer Research Institute. The NCRI, which began work in 2001, is a UK–wide partnership between government, charity and industry, which promotes cooperation in cancer research among 20 member organizations for the benefit of patients, the public and the scientific community. Current members of NCRI [54] have all demonstrated that they have both annual cancer research expenditure in the United Kingdom in excess of £1 million and also an appropriate peer–review system for ensuring the scientific quality of the research they fund.

The NCRI members are obliged to provide full, publishable details of the cancer research they fund to the NCRI for inclusion in the NCRI Cancer Research Database and to contribute to the development and implementation of cancer research strategies in the UK.

The activities of NCRI are led by the NCRI Board. The NCRI Secretariat carries out the Board’s decisions and generally helps members to interact. All NCRI activities are overseen by the NCRI Board, which makes collective decisions on behalf of all the NCRI members. The Board does not itself allocate funds. When the Board agrees upon a new activity, individual members are invited to participate and to contribute funding.

Because the member organizations all have different missions, this leads to different partnership groupings for the range of activities promoted. Members of the Board are drawn from senior staff in the partner organizations, patient representatives and others involved in NCRI’s work. The Chair is rotated every 2–3 years, and there is periodic rotation of some seats to ensure that all partners have the opportunity to serve at some stage.

The NCRI helps with joint planning and coordination among its members and focuses on areas where progress could not be achieved by a single member organization on its own. Specifically, the NCRI:

Maintains a Cancer Research Database and undertakes analyses of what research is being done – this informs decisions about new research.
Develops research initiatives on specific topics, to which a group of members will contribute (e.g. programmes in prostate cancer or in cancer prevention).
Helps to coordinate clinical trials and experimental cancer medicine research within networks throughout the United Kingdom.
Develops facilities and resources for research (e.g. the collection of tumour samples or trying to make the management of research data more effective, i.e. informatics).

### 11.3. European Molecular Biology Organization—EMBO (www.embo.org)

The EMBO is a European success story. The EMBO was created in 1964 by a group of scientists who recognized that to promote the development of molecular biology in Europe there was a need for a formal structure based on excellence in science.

The EMBO had two initial goals: the creation of a central molecular biology laboratory and the establishment of networking activities to enhance interactions between European laboratories.

The EMBO’s networking activities started with the provision of fellowships and the election of 200 biologists as the organization’s first members.

The founding members received a five–year grant from the Volkswagen Foundation, and under the presidency of Raymond Appleyard, who devoted half of his time to EMBO, EMBO activities grew using private funds and dedicated members to execute its tasks.

Long–term funding and support for EMBO’s actions came in 1969 with the establishment of the European Molecular Biology Conference (EMBC), an intergovernmental body created solely for the purpose of funding EMBO. EMBC agreed to fund the EMBO and give the EMBO full freedom on the condition of having full insight into the organization. EMBC has since grown to 25 Member States including many countries in the European Union and beyond.

After an additional five years, EMBC accepted EMBO’s proposal to establish an European laboratory, and the European Molecular Biology Laboratory (EMBL) was founded in 1974. It is supported by 18 Member States including nearly all of Western Europe and Israel, and consists of the Main Laboratory in Heidelberg, Germany, and four Outstations.

The EMBO activities expanded over the decades and today EMBO has approximately 1200 members in Europe and 100 associate members worldwide, including a number of Nobel Prize winners (2007 Nobel Prize in Physiology or Medicine, Sir Martin J. Evans is a member of EMBO). The cornerstone of the organization is the EMBO Council, consisting of 15 members elected for a three–year term and of chairs of EMBO Committees and representatives of EMBC.

Committees are responsible for the implementation of policy and advice from the Council. EMBO headquarters are located in Heidelberg, Germany, and have a staff of around 50 employees under the direction of an Executive Director and a Secretary General. The EMBL has 130 staff employees and around 400 post–docs.

The EMBO activities are funded predominantly by the European Molecular Biology Conference. The organization runs an extended programme of activities ranging from fellowships, courses and workshops, to science and society activities and scientific publications.

EMBO nurtures training and careers at all stages of the scientific career path through courses, workshops conferences and fellowships – and most recently through the Young Investigator Programme, which promotes Europe’s best young scientists in the early stages of their research careers.

The EMBO promotes mobility throughout Member States and globally through post–doctoral and short–term fellowships. On completion of their fellowship the majority of EMBO post–doctoral fellows return to their home countries transferring new skills and contacts to their respective Member States. Short–term fellows return to their countries with practical techniques having built a strong foundation for future scientific collaboration.

The EMBO’s World Activities promote international scientific cooperation and bridge technological differences to further research. Jointly created by EMBO and the European Commission, The Life Sciences Mobility Portal offers a series of searchable databases pointing life scientists to sources of funding, training, and employment.

The Courses and Workshops Programme has proved itself to be an essential part of EMBO activities since its establishment in the 1960s. Through this programme, funds are provided annually for practical courses, lecture courses, workshops and conferences globally. Close links are also maintained between the scientific community and a wider audience through science and society activities for educators, students, policy makers, industry and the media.

A boom in electronic data means that biologists are increasingly going online to search for literature and diverse data types. Through integrating and organizing online information EMBO’s Electronic Information Programme turns information overload into information management. The EMBO Journal, its sister publications EMBO reports and Molecular Systems Biology provide a variety of formats to cover topics related to biology at a molecular level.

### 11.4. European Strategy Forum on Research Infrastructures—ESFRI

The ESFRI was launched in April 2002 (http://cordis.europa.eu/esfri/). It consists of representatives of EU Member States and Associated States, appointed by Ministers In Charge of Research, and one representative of the European Commission.

The role of ESFRI is to support a coherent approach to policy making on research infrastructures in Europe and to act as an incubator for international negotiations about concrete initiatives.

#### ESFRI—Mode of operation

The European Strategy Forum on Research Infrastructures was established following a recommendation from the EU Council. Its main scope is to:

support a coherent and strategy–led approach to policy making on research infrastructures in Europe;
facilitate multilateral initiatives leading to the better use and development of research infrastructures.

The Forum acts as an informal body on issues raised by one or more country’s delegations. The Strategy Forum gives national authorities the opportunity to be informed of and to explore (i.e. without ‘*a priori*’ commitments) international and national initiatives concerned with the building or upgrading of research infrastructures of European significance. ESFRI therefore acts as an incubator for pan–European research infrastructures.

For its internal operation, the Forum is divided into several organs.

#### ESFRI Structure

RWG: Roadmap Working Group
PSE: Physical Science and Engineering
BMS: Biological and Medical Science
SSH: Social Science and Humanities
ENV: Environmental

*Expert Groups: only if appropriate

#### ESFRI Membership, Chair and Secretariat

European Strategy Forum on Research Infrastructures brings together one or two senior science policy officials, representing the EU Member and Associated States and a senior science policy official of the European Commission. The members of each delegation are nominated by their Minister(s) for two years and may be reconfirmed whenever appropriate. The ESFRI Secretariat is provided by the Commission services (Unit Research Infrastructures).

#### Executive Board

The Executive Board is composed of the ESFRI Chair, the Commission representative and three ESFRI members selected by consensus. The selected board members are designated by the Forum for a two–year non–renewable term. For the preparation of the European Roadmap, the Executive Board is supported by the Roadmap Working Group Chairs.

### 11.5. Innovative Medicines Initiative—IMI

The IMI project (www.imi-europe.org) is a JTI under Article 171. On 20 December 2007, the European Council adopted the regulation establishing the IMI Joint Undertaking.

Having once been the world leader in pharmaceutical research, Europe is now lagging in both public and private research investment. The Innovative Medicines Initiative Joint Technology Initiative aims to improve this situation by a unique collaboration with the pharmaceutical sector. For the first time, competing pharmaceutical companies will collaborate on research to improve the drug development process. Participation of academia and clinical centres, small and medium sized enterprises (SMEs), patient organizations and public authorities (including regulators) will be essential and will lead to faster uptake of results.

Traditional EU collaborative research instruments cannot achieve the coordination of research efforts necessary to cope with the scale and complexity of the research challenges involved. The JTI, introduced in the Seventh Framework Programme (FP7) as a new way of realising public––private partnerships in research at the European level, is the most appropriate means of coordinating these efforts given the scale and complexity of the research challenges. Therefore, the Commission proposed the implementation of the Strategic Research Agenda on Innovative Medicines as a JTI in its proposal for FP7, confirmed through the co–decision by the Council and the European Parliament.

A Joint Undertaking (JU) between the European Federation of Pharmaceutical Industries and Associations (EFPIA) and the European Commission will be the legal entity responsible for implementing the JTI (Council Regulation).

Competencies and resources from the private and the public sectors will be pooled in a public––private partnership. The IMI Joint Undertaking will be set up as a Community body by a Council Regulation under Article 171 of the Treaty. It will have a total budget of 2 billion Euros: half contributed by the EC from FP7, half contributed by EFPIA and the research–based pharmaceutical companies that are full members of EFPIA

The IMI Joint Undertaking will support research activities conducted in the Member States and in the countries associated with FP7, following open calls for proposals. The Community contribution will be used exclusively to support academia and clinical centres, SMEs, patient organizations and public authorities (including regulators). The European Federation of Pharmaceutical Industries and Associations member companies will carry the costs of their part of the research collaboration at a value equal to the Community contribution. The governance structure for IMI, developed through a close collaboration between the Commission and the European Federation of Pharmaceutical Industries and Associations, comprises the Joint Undertaking, the Member States Group and the Stakeholder Forum and clearly reflects the public–private nature of the initiative. In their role as founders, the Commission and EFPIA will share equally the responsibilities and costs for the implementation of the IMI Joint Technology Initiative. It will be governed by the IMI Joint Undertaking (Board, Executive Office and Scientific Committee) and by two additional groups (Member States Group and Stakeholder Forum).

The IMI Joint Undertaking will manage the implementation of the research activities outlined in the Research Agenda. The Executive Office, with its independent staff, will be responsible for the day–to–day management, including the call and evaluation process, grant agreements. The Board, composed of the founding members, will have overall responsibility for the operations of the IMI Joint Undertaking and decide on the annual implementation of the research activities, following consultation with the Scientific Committee. It will also be responsible for communication and coordination between IMI and Member State activities (via the Member States Group). A Stakeholder Forum will be held annually to exchange views on the ongoing or planned research activities.

The IMI Joint Undertaking should be considered as a Community body and be established for a period ending on 31 December 2017. It will have its seat in Brussels, Belgium. On 20 December 2007, the European Council adopted regulations establishing the joint technology initiatives (JTIs) aimed at implementing the research project—Innovative Medicines Initiative (IMI)—intended to strengthen the European pharmaceutical sector by accelerating the development of safe and more effective medicines for patients. The first calls for innovative research projects are expected for 2008.

### 11.6. Coordination of Cancer Clinical Practice Guidelines in Europe (CoCanCPG project)

Oncology practice guidelines have shown their potential to improve evidence–based practices and outcome of patients with cancer. The goal of the CoCanCPG project, www.cocancpg.eu, is to foster equitable access to high–quality cancer care in Europe through cooperation in guideline development and research.

CoCanCPG is a Coordinated Action under the ERA–Net Scheme, which has received funding for four years under the 6th Framework Programme of the European Commission. The project started in February 2006. The CoCanCPG consortium consists of 17 partners from nine European countries (Belgium, France, Germany, Hungary, Italy, Lithuania, Spain, The Netherlands, the United Kingdom), Israel and Canada.

It answers to the current challenges encountered by guideline programmes: reducing duplication of effort and overcoming fragmentation in guideline development and research. This is being achieved through the implementation of innovative approaches to foster the timely and pertinent translation of new knowledge into improved care for patients with cancer.

Thus, CoCanCPG undertakes the following:

implementing a common framework for sharing knowledge, methods and skills;
setting up joint activities for guideline development;
assembling a critical mass for pertinent research into guideline methods;
creating an appropriate and sustainable environment for sharing and joint activities.

The Consortium has successfully completed a benchmarking of practices as a basis for the development of common management approaches and setting up joint activities at the transnational level. This is the first time that a formal benchmarking has been conducted among CPG programmes from different countries and organizational backgrounds. CoCanCPG will further contribute to the European Research Area (ERA) in the field of cancer research by identifying clinical research priorities on the basis of areas where no evidence exists currently.

### 11.7. Other examples of European cooperation

There are other examples of inter–governmental agreements to promote scientific developments in Europe, e.g. The European Organization for Nuclear Research (CERN), created in 1954 and The IMI Joint Undertaking will manage the implementation of the research activities outlined in the Research Agenda. The Executive Office, with its independent staff, will be responsible for the day–to–day management, including the call and evaluation process, grant agreements. The Board, composed of the founding members, will have overall responsibility for the operations of the IMI Joint Undertaking and decide on the annual implementation of the research activities following consultation with the Scientific Committee. It will also be responsible for communication and coordination between IMI and Member State activities (via the Member States Group). A Stakeholder Forum will be held annually to exchange views on the ongoing or planned research activities.

The IMI Joint Undertaking should be considered as a Community body and be established for a period ending on 31 December 2017. It will have its seat in Brussels, Belgium. On 20 December 2007, the European Council adopted regulations establishing the JTIs aimed at implementing the research project—IMI—intended to strengthen the European pharmaceutical sector by accelerating the development of safe and more effective medicines for patients. The first calls for innovative research projects are expected for 2008.

## Glossary

6th FP/FP6: Sixth Framework Programme
7th FP/FP7: Seventh Framework Programme
BOF: Board of Funders
CCC: Comprehensive Cancer Centre
CERN: European Organization for Nuclear Research
COCANCPG: Coordination of Cancer Clinical Practice Guidelines in Europe
DD: Drug Discovery
EACR: European Association for Cancer Research
EATRIS: European Advanced Translational Infrastructure in Medicine
ECCO: European Cancer Organization
ECDC: European Centre for Disease Control
ECI: European Cancer Initiative
ECRIN: European Clinical Research Infrastructure Network
EFPIA: European Federation of Pharmaceutical Industries and Association
EFSA: European Food Safety Agency
EMBC: European Molecular Biology Conference
EMBL: European Molecular Biology Laboratory
EMBO: European Molecular Biology Organisation
EMEA: European Medicines Agency
EO: Executive Office
EORTC: European Organisation for Research and Treatment of Cancer
ERA: European Research Area ERANET European Research Area Network
ERC: European Research Council
ESA: External Stakeholders Assembly
ESFRI: European Strategy Forum on Research Infrastructures
EU: European Union
IARC: International Agency for Research on Cancer
IMI: Innovative Medicines Initiative
JTI: Joint Technology Initiative
JU: Joint Undertaking
MD: Doctor of Medicine (clinician)
MSc: Master of Science
US–NCI: National Cancer Institute (USA)
NCRI: National Cancer Research Institute (UK)
OECI: Organisation of European Cancer Institutes
PhD: Doctor of Philosophy (researcher)
R&D: Research and Development
SAB: Scientific Advisory Board
SC: Standing Committee
SEER: Surveillance Epidemiology and Results
SEMM: European School of Molecular Medicine
SME: Small and Medium Enterprises
SRA: Strategic Research Agenda
TC: Temporary Committees
TR: Translational Research
TUBAFROST: The European Human Frozen Tissue Bank
